# Reliability Inference in Constant Stress Accelerated Life Tests Based on the Exponentiated Half-Logistic Model

**DOI:** 10.3390/e28101140

**Published:** 2026-10-16

**Authors:** Tuğçe Hancı, Fatma Gül Akgül

**Affiliations:** 1 Graduate School of Natural and Applied Science, Karadeniz Technical University, 61080 Trabzon, Türkiye; hancitugce61@gmail.com; 2 Department of Computer Science, Faculty of Science, Karadeniz Technical University, 61080 Trabzon, Türkiye

**Keywords:** constant stress accelerated life testing, exponentiated half-logistic distribution, reliability function, mean time to failure function, efficiency, Monte Carlo simulation

## Abstract

Accelerated life testing is an efficient methodology that enables the acquisition of failure time data in a shorter period by subjecting test units to different stress levels, compared to tests conducted under normal operating conditions. In this study, it is assumed that the lifetime of a product under a constant stress level follows an exponentiated half-logistic distribution and the model parameters, reliability function and mean time to failure function are estimated using eleven different frequentist methods, namely, maximum likelihood, least squares, weighted least squares, Cramér–von Mises, Anderson–Darling, right-tail Anderson–Darling, left-tail Anderson–Darling, second-order left-tail Anderson–Darling, maximum product of spacings, minimum spacing absolute distance, and minimum spacing absolute-log distance. The performances of the estimators are compared through an extensive Monte Carlo simulation study. Furthermore, a real dataset is analyzed to evaluate the practical effectiveness of the proposed methods.

## 1. Introduction

In the current global competitive environment, ensuring the long-term and reliable performance of products and components is critical, and demand for products with a low probability of failure has increased substantially. However, the collection of failure time data for products and components under normal operating conditions is generally avoided in practice due to the high cost and time-consuming nature of the process. To overcome this problem, tests are conducted at various stress levels in order to obtain failure time data for the tested units within a shorter period of time, see [1]. These tests are called accelerated life testing (ALT). In an ALT experiment, the tested units are evaluated by subjecting them to stress types, such as temperature, humidity, pressure, and voltage at levels higher than those under normal operating conditions, see [2,3]. In ALT models stress factors can be applied using various approaches, including constant stress ALT (CSALT), step-stress (simple or multiple), random stress, progressive stress, sinusoidal-cyclic stress, and square-waves cyclic stress; for more detailed information see [1]. Among them, CSALT is widely preferred due to its simplicity, ease of modeling, and ability to provide reliable estimates. Although longer test durations are required to obtain sufficient failure data at low stress levels, the data obtained under the CSALT yield higher accuracy and consistency in reliability estimation compared to those obtained under complex stress profiles. A comprehensive review of CSALT models is provided by [4].

In the context of the CSALT model, inferences based on complete data under different lifetime distributions using various classical estimation methods have been extensively investigated by numerous researchers. In the majority of existing studies, within the CSALT framework, either the maximum likelihood (ML) method has been considered exclusively, or approaches combining ML with least squares (LS), weighted least squares (WLS) estimators, and probability-weighted moments (PWM) have been adopted. In this context, readers may refer to the works of [5,6,7,8,9,10,11,12]. More recently Abdel Ghaly et al. [13] considered the estimation of CSALT model parameters using the ML, LS, WLS, and PWM methods when the underlying distribution is exponentiated inverse Weibull. Wang and Shi [14] estimated the model parameters of the generalized half-normal distribution under the CSALT and constructed confidence intervals based on asymptotic theory and bootstrap techniques. Nassar and Dey [15] employed six different estimation methods to estimate the model parameters of CSALT and the reliability function for the exponentiated Rayleigh distribution. Xin et al. [16] considered the estimation of CSALT model parameters by using ML and Bayesian methods when the underlying distribution is doubly truncated three-parameter Burr XII. Dey and Nassar [17,18] employed different methods to estimate the unknown CSALT model parameters and the reliability function of the generalized inverse exponential and exponentiated Lindley distributions, respectively. Nassar et al. [19] derived the estimators of CSALT model parameters as well as the reliability function and the mean time to failure (MTTF) function based on eight different estimation methods under the assumption that the lifetime data follow an exponentiated Poisson exponential distribution. Kumar et al. [20,21] employed nine different methods to estimate the parameters of the CSALT model for the generalized inverse Lindley and the generalized power Lindley distributions, respectively. Nassar et al. [22] investigated the estimation of exponentiated exponential parameters under CSALT model by using different estimation methods. Du and Gui [23] considered the estimation of model parameters of CSALT when the underlying distribution is Gompertz. Newer [24] developed a statistical model for multiple CSALT, combining ranked set sampling with progressive Type-II censoring under exponentiated exponential distribution to obtain parameter estimates both frequentist and Bayesian approaches. Osman et al. [25] considered CSALT under Type-II censored data and the assumption that failure times at each stress level follow the Gull alpha power Fréchet distribution, used ML approach to derive point and interval estimates for the model parameters, acceleration factor, reliability and hazard functions. McGrath and Burke [26] proposed a simulation-based approach for determining optimal test plans in CSALT with multiple design points. Akgül [27] obtained the model parameters using different estimation methods, assuming that the product lifetime follows the exponentiated Pareto distribution under CSALT.

The choice of the lifetime distribution is particularly important in ALT because the distribution must be sufficiently flexible to represent the failure–time behavior observed at different stress levels. The exponentiated half-logistic (EHL) distribution is attractive in this setting because its shape parameter can substantially alter both the density and hazard-rate behavior. Consequently, the EHL model can represent failure–time patterns that may not be adequately described by more restrictive lifetime distributions. However, the literature reviewed above does not provide a systematic comparison of a broad set of classical estimation criteria for the EHL distribution within the CSALT framework. In particular, there is limited evidence on how likelihood-based, LS, goodness-of-fit-based, and spacing-based estimators compare simultaneously for the estimation of the CSALT parameters and derived reliability measures.

Accordingly, the purpose of this study is not to introduce a new estimation criterion, but to provide a systematic comparative investigation of existing frequentist estimators when they are applied to the EHL-CSALT model. This distinction is important because the contribution of the study is comparative and application-oriented: it examines whether conclusions about estimator performance depend on the estimation criterion, sample size, stress-related parameter settings, and the target quantity being estimated.

More specifically, the study makes the following contributions. First, eleven classical estimation methods are formulated and compared under a common EHL-based CSALT model: ML, LS, WLS, Cramér–von Mises (CM), Anderson–Darling (AD), right-tail Anderson–Darling (RTAD), left-tail Anderson–Darling (LTAD), Anderson–Darling left-tail second order (ADLTS), maximum product of spacings (MPSs), minimum spacing absolute distance (MSAD), and minimum spacing absolute-log distance (MSALD). Second, their finite-sample behavior is examined for the model parameters, the normal-use shape parameter, the reliability function, and the MTTF, rather than evaluating parameter estimates alone. Third, a Monte Carlo study is used to investigate the effects of sample size and stress–life parameter settings on estimation accuracy. Fourth, the methods are applied to a real accelerated-life dataset from metal–oxide–silicon (MOS) capacitors to examine their practical behavior across multiple temperature stress levels. Taken together, these analyses provide an empirical basis for understanding the relative behavior of established estimators in the EHL-CSALT setting.

The remainder of this paper is organized as follows. Section 2 introduces the CSALT model for the EHL distribution, along with its fundamental formulation and underlying assumptions. Section 3 provides a detailed description of the eleven estimation methods considered under the CSALT framework for the EHL distribution. In Section 4, a Monte Carlo simulation study is conducted to evaluate the comparative performance of each estimation method and examine the effectiveness of the proposed model. Section 5 demonstrates the applicability of the EHL distribution using a real dataset corresponding to three different stress levels. Finally, Section 6 presents concluding remarks and general discussions based on the obtained results.

## 2. Model Assumptions

The EHL distribution was first introduced by Kang and Seo [28] as a flexible statistical model for lifetime data. Since then, it has been widely applied across various fields, including insurance, engineering, medicine, and education, see [29]. In recent years, the EHL distribution has been commonly used in reliability studies. For example, Rao et al. [30] developed a multiple deferred state sampling plan for a time-truncated life test under the assumption that the product lifetime follows the EHL distribution. Alotaibi et al. [31] proposed a bivariate EHL distribution with EHL marginal distributions and investigated several statistical properties of the proposed model, including its product moments as well as marginal and conditional distributions. Rao et al. [32] introduced a multiple dependent state repetitive sampling plan under the assumption that product lifetimes follow the EHL distribution. Hassan and Morgan [33] examined the estimation of stress-strength reliability in the presence of outliers when the stress and strength random variables follow the EHL distribution.

Let X be a random variable following the EHL distribution with shape and scale parameters α and β, respectively. Then, the probability density function (pdf), cumulative distribution function (cdf), reliability function and hazard rate function (hrf) are given by the following expressions:

(1) $$f\left(x;\alpha ,\beta \right)=\frac{2\alpha \beta e^{-\beta x}}{{\left(1+e^{-\beta x}\right)}^{2}}{\left(\frac{1-e^{-\beta x}}{1+e^{-\beta x}}\right)}^{\alpha -1},x>0,\;\alpha ,\beta >0,$$

(2) $$F\left(x;\alpha ,\beta \right)={\left(\frac{1-e^{-\beta x}}{1+e^{-\beta x}}\right)}^{\alpha },x>0,\alpha ,\beta >0,$$

(3) $$R\left(x;\alpha ,\beta \right)=1-{\left(\frac{1-e^{-\beta x}}{1+e^{-\beta x}}\right)}^{\alpha },x>0,\alpha ,\beta >0,$$

(4) $$h\left(x;\alpha ,\beta \right)=\frac{2\alpha \beta e^{-\beta x}{\left(\frac{1-e^{-\beta x}}{1+e^{-\beta x}}\right)}^{\alpha }}{\left(1-e^{-2\beta x}\right)\left[1-{\left(\frac{1-e^{-\beta x}}{1+e^{-\beta x}}\right)}^{\alpha }\right]},x>0,\alpha ,\beta >0,$$

respectively. It should be stated that the shape parameter α plays a crucial role in determining the density and reliability characteristics of the distribution. The behavior of the distribution varies significantly across different values of α. For α≤1, the distribution exhibits a monotonically decreasing pattern, whereas for α>1, it assumes a right-skewed unimodal structure. The scale parameter β controls the spread of the distribution along the time axis and influences of system degradation dynamics. As a special case, when α=1, the EHL distribution reduces to the half-logistic distribution, see [34].

**Figure 1:**
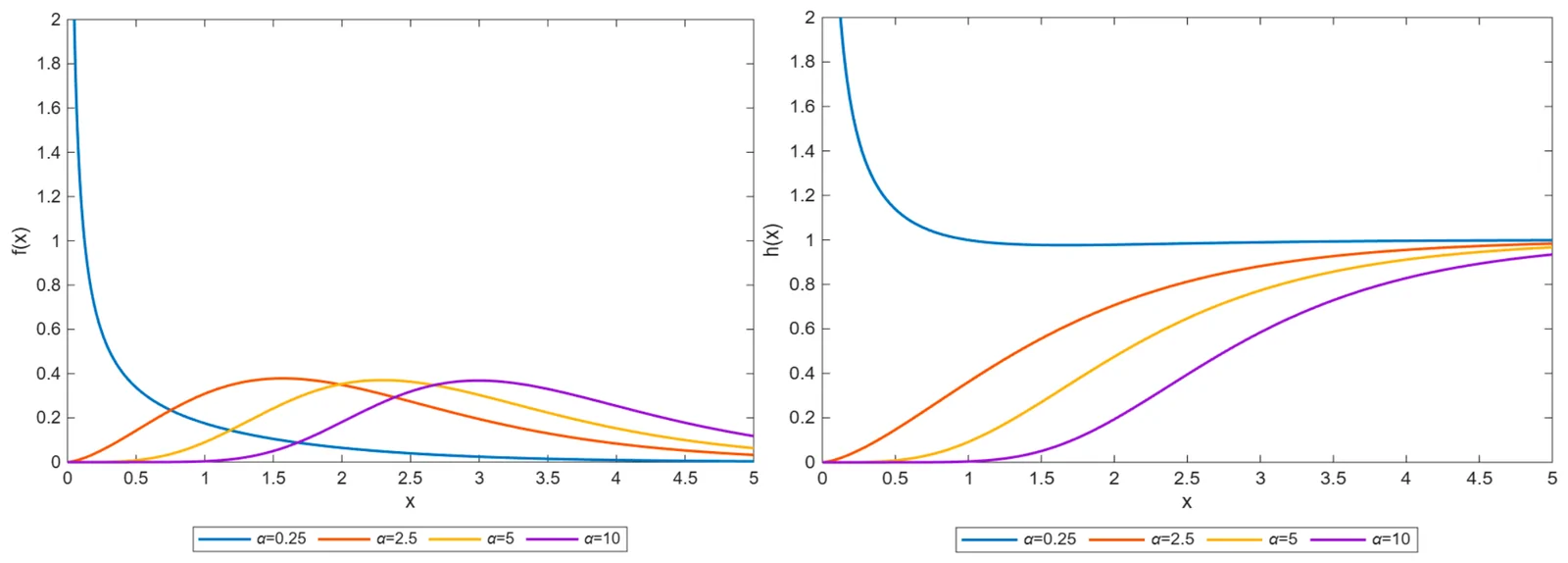
Pdf and hrf plots of the EHL distribution under varying shape parameter values.

Figure 1 presents the pdf and hrf of the EHL distribution for selected values of the shape parameter α. The results show that the distribution is highly sensitive to this parameter. For small α, the pdf is strongly right-skewed with mass concentrated near zero, indicating early-life failures. As α increases, the density becomes unimodal and shifts to the right, reflecting delayed failure behavior. The hrf exhibits distinct patterns depending on α. Specifically, it is decreasing for small values of α, while it becomes increasing for larger values, reflecting aging effects. In all cases, as time increases, the hrf converges to the parameter β, indicating a stabilization at a constant failure rate level. These findings confirm that the EHL distribution is sufficiently flexible to accommodate various failure rate structures, making it suitable for reliability and ALT applications.

MTTF function is described as the average time that a system will function prior to failure, and for EHL distribution the MTTF function is calculated as given below:

(5) $$MTTF=\int_{0}^{\infty }R\left(t\right)dt=\int_{0}^{\infty }tf\left(t\right)dt=\int_{0}^{\infty }t\frac{2\alpha \beta e^{-\beta x}}{{\left(1+e^{-\beta x}\right)}^{2}}{\left(\frac{1-e^{-\beta x}}{1+e^{-\beta x}}\right)}^{\alpha -1}dt=\frac{1}{\beta }\left(\gamma \left(\frac{\alpha +1}{2}\right)-\gamma \left(\frac{1}{2}\right)\right).$$

We make the following assumptions for estimation under CSALT for EHL distribution.

1. A total of *N* identical test units are allocated into *k* distinct groups, where the jth group consists of $n_{j}$ units. Accordingly, the overall sample size satisfies $N=\sum_{j=1}^{k}n_{j}.$
2. The experiment is carried out under *k* accelerated stress levels $S_{j},\;j=1,2,\ldots ,k,$ in addition to the normal operating stress denoted by $S_{u}$. These stress levels are ordered increasingly such that $S_{u}<S_{1}<\cdots <S_{k}$. For each j=1,…k, $n_{j}$ test units are subjected to a constant stress level $S_{j}$.
3. The observed failure times $X_{ij},\;i=1,2,\ldots ,n_{j};j=1,\ldots ,k$ are assumed to be independent and identically distributed (i.i.d.) random variables following the EHL distribution, with pdf given in (1).
4. The shape parameter $\alpha_{j}$ of the lifetime distribution at the jth stress level is assumed to depend on the applied stress $S_{j}$ through a log-linear relationship, expressed as $\ln\alpha_{j}=a+bS_{j}$ or equivalently, $\alpha_{j}=\exp\left(a+bS_{j}\right)$ where $\alpha_{j}$ represents the shape parameter corresponding to the lifetime distribution under stress level $S_{j}$, while a and b are unknown parameters. This formulation guarantees the positivity of $\alpha_{j}$ and provides a simple representation of the stress effect on the distributional shape. The usage of a log-linear stress relationship is consistent with previous CSALT studies, including [13,15,17,18] and is adopted here as an established modeling formulation rather than as a new stress–life relationship.
5. The scale parameter is assumed to be common across the accelerated stress levels, such that $\beta_{j}=\beta >0$, for j=1,…,k. Thus, the stress effect is modeled through the shape parameter, while β represents a common scale parameter.

## 3. Estimation of the Unknown Parameters

In this study, eleven different estimation methods are considered in order to provide a comprehensive comparison under the CSALT model with the EHL distribution. These methods are chosen due to their widespread use in reliability analysis and their different sensitivity structures with respect to distribution tails, parameter bias, and data dispersion. In particular, likelihood-based methods (ML and MPS) are known for their asymptotic efficiency, while least-squares-based methods (LS and WLS) provide computational simplicity. Goodness-of-fit-based estimators (AD, CM, RTAD, LTAD, ADLTS) are included due to their ability to emphasize different regions of the distribution, especially tail behavior, which is critical in reliability applications. Additionally, modified deviation-based methods (MSAD and MSALD) are considered to improve robustness against outliers and model misspecifications.

Therefore, the inclusion of these methods allows for a comprehensive assessment of estimation performance under different statistical characteristics, ranging from likelihood-based inference to distance-based and robustness-oriented approaches.

### 3.1. Method of Maximum Likelihood

In this subsection, the ML estimators of the parameters of EHL distribution based on CSALT are obtained. By using (1), the likelihood function can be written as follows:

$$L\left(a,b,\beta ,x\right)=\prod_{j=1}^{k}\prod_{i=1}^{n_{j}}\frac{2e^{a+bS_{j}}\beta e^{-\beta x_{ij}}}{{\left(1+e^{-\beta x_{ij}}\right)}^{2}}{\left(\frac{1-e^{-\beta x_{ij}}}{1+e^{-\beta x_{ij}}}\right)}^{e^{a+bS_{j}}-1}.\;$$

By taking the natural logarithm of the likelihood function, we derive the log-likelihood function as given below:

$$\begin{array}{cc} l\left(a,b,\beta \right) & =Nln2+\sum_{j=1}^{k}\sum_{i=1}^{n_{j}}\left(a+bS_{j}\right)+Nln\beta -\beta \sum_{j=1}^{k}\sum_{i=1}^{n_{j}}x_{ij}\;\\ & +\sum_{j=1}^{k}\sum_{i=1}^{n_{j}}\left(e^{a+bS_{j}}-1\right)ln\left(\frac{1-e^{-\beta x_{ij}}}{1+e^{-\beta x_{ij}}}\right)-2\sum_{j=1}^{k}\sum_{i=1}^{n_{j}}\ln\left(1+e^{-\beta x_{ij}}\right). \end{array}$$

The ML estimators of the unknown parameters a,b and β are obtained by solving the three likelihood equations simultaneously. The detail expressions of the log-likelihood equations are given in Appendix A. It is obvious that these equations do not have closed-form solutions, therefore numerical techniques are employed to obtain the ML estimators of the parameters of interest. The ML estimators of a, b, and β are denoted by ${\hat{a}}_{ML},{\hat{b}}_{ML}$ and ${\hat{\beta }}_{ML}$, respectively. The corresponding ML estimator of the shape parameter $\alpha_{j},j=1,\ldots ,k$ is expressed as follows:

$${\hat{\alpha }}_{MLj}=e^{{\hat{a}}_{ML}+{\hat{b}}_{ML}S_{j}}.$$

### 3.2. Method of Least Squares

Let $x_{11:N},\ldots ,x_{n_{k}k:N}$ be the order statistics of a random sample of size N from the EHL distribution under CSALT. The LS estimators of a, b, and β, say ${\hat{a}}_{LS},\;{\hat{b}}_{LS}$ and ${\hat{\beta }}_{LS}$, are obtained by minimizing the following function, see [35]:

$$S\left(a,b,\beta \right)={\sum_{j=1}^{k}\sum_{i=1}^{n_{j}}\left[{\left(\frac{1-e^{-\beta x_{ij:N}}}{1+e^{-\beta x_{ij:N}}}\right)}^{e^{a+bS_{j}}}-\frac{i}{n_{j}+1}\right]}^{2}.$$

From that part, we use $x_{ij}$ instead of $x_{ij:N}$ for the sake of simplicity. The LS estimators are derived by solving the corresponding non-linear equations given in Appendix B. Now the LS estimator of $\alpha_{j},\;j=1,\ldots ,k$ can be obtained as follows:

$${\hat{\alpha }}_{LSj}=e^{{\hat{a}}_{LS}+{\hat{b}}_{LS}S_{j}}.$$

### 3.3. Method of Weighted Least Squares

The WLS estimators of the unknown parameters a,b and β, namely ${\hat{a}}_{WLS},\;{\hat{b}}_{WLS}$ and ${\hat{\beta }}_{WLS}$, are derived by minimizing the following function, see [36]:

$$W\left(a,b,\beta \right)={\sum_{j=1}^{k}\sum_{i=1}^{n_{j}}\frac{{\left(n_{j}+1\right)}^{2}\left(n_{j}+2\right)}{i\left(n_{j}-i+1\right)}\left[{\left(\frac{1-e^{-\beta x_{ij}}}{1+e^{-\beta x_{ij}}}\right)}^{e^{a+bS_{j}}}-\frac{i}{n_{j}+1}\right]}^{2}.$$

The estimators of a,b and β are obtained by solving the corresponding non-linear equations given in Appendix C. The WLS estimators of $\alpha_{j},\;j=1,\ldots ,k$ are obtained as follows:

$${\hat{\alpha }}_{WLSj}=e^{{\hat{a}}_{WLS}+{\hat{b}}_{WLS}S_{j}}.$$

### 3.4. Method of Cramer-Von Mises

The CM estimators of the unknown parameters a,b and β, namely ${\hat{a}}_{CM},\;{\hat{b}}_{CM}$ and ${\hat{\beta }}_{CM}$, are derived by minimizing the following function with respect to parameters of interest, see [37]:

$$C\left(a,b,\beta \right)=\frac{1}{12N}+\sum_{j=1}^{k}\sum_{i=1}^{n_{j}}{\left[{\left(\frac{1-e^{-\beta x_{ij}}}{1+e^{-\beta x_{ij}}}\right)}^{e^{a+bS_{j}}}-\frac{2i-1}{2n_{j}}\right]}^{2}.$$

The estimators of a,b and β are obtained from the solution of the corresponding non-linear equations given in Appendix D. The CM estimators of $\alpha_{j},\;j=1,\ldots ,k$ are obtained as follows:

$${\hat{\alpha }}_{CMj}=e^{{\hat{a}}_{CM}+{\hat{b}}_{CM}S_{j}}.$$

### 3.5. Method of Anderson Darling

The AD estimators for the unknown parameters, ${\hat{a}}_{AD},{\hat{b}}_{AD}$ and ${\hat{\beta }}_{AD}$, are derived by minimizing the following function:

$$A\left(a,b,\beta \right)=-N-\sum_{j=1}^{k}\sum_{i=1}^{n_{j}}\frac{2i-1}{n_{j}}\left[lnF\left(x_{ij};a,b,\beta \right)\right.+\left.\ln\left(1-F\left(x_{n_{j}+1-ij};a,b,\beta \right)\right)\right].$$

The estimators of a,b and β are obtained by solving the corresponding non-linear equations given in Appendix E. The AD estimators of $\alpha_{j},\;j=1,\ldots ,k$ are obtained as follows:

$${\hat{\alpha }}_{ADj}=e^{{\hat{a}}_{AD}+{\hat{b}}_{AD}S_{j}}.$$

### 3.6. Method of Right-Tail Anderson Darling

The RTAD estimators of the unknown parameters a,b and β, namely ${\hat{a}}_{RTAD},\;{\hat{b}}_{RTAD}$ and ${\hat{\beta }}_{RTAD}$, are derived by minimizing the following function, see [38]:

$$S\left(a,b,\beta \right)={\sum_{j=1}^{k}\sum_{i=1}^{n_{j}}\left[{\left(\frac{1-e^{-\beta x_{ij:N}}}{1+e^{-\beta x_{ij:N}}}\right)}^{e^{a+bS_{j}}}-\frac{i}{n_{j}+1}\right]}^{2}.$$

The estimators of a,b and β are obtained from the solution of the corresponding non-linear equations given in Appendix F. The RTAD estimators of $\alpha_{j},\;j=1,\ldots ,k$ are obtained as follows:

$${\hat{\alpha }}_{RTADj}=e^{{\hat{a}}_{RTAD}+{\hat{b}}_{RTAD}S_{j}}.$$

### 3.7. Method of Left-Tail Anderson Darling

The LTAD estimators of unknown parameters, namely ${\hat{a}}_{LTAD},\;{\hat{b}}_{LTAD}$ and ${\hat{\beta }}_{LTAD}$, are derived by minimizing the following function:

$$LT\left(a,b,\beta \right)=-\frac{3}{2}N+2\sum_{j=1}^{k}\sum_{i=1}^{n_{j}}F\left(x_{ij};a,b,\beta \right)-\sum_{j=1}^{k}\sum_{i=1}^{n_{j}}\frac{2i-1}{n_{j}}\ln\left(F\left(x_{ij};a,b,\beta \right)\right).$$

The estimators of the parameters of interest are obtained by solving the corresponding non-linear equations given in Appendix G. The LTAD estimator of $\alpha_{j},\;j=1,\ldots ,k$ are obtained as follows:

$${\hat{\alpha }}_{LTADj}=e^{{\hat{a}}_{LTAD}+{\hat{b}}_{LTAD}S_{j}}.$$

### 3.8. Method of Anderson Darling Left-Tail Second Order

The ADLTS estimators of the unknown parameters a,b and β, namely ${\hat{a}}_{ADLTS},\;{\hat{b}}_{ADLTS}$ and ${\hat{\beta }}_{ADLTS}$, are derived by minimizing the following function:

$$LTS\left(a,b,\beta \right)=2\sum_{j=1}^{k}\sum_{i=1}^{n_{j}}\ln\left(F\left(x_{ij};a,b,\beta \right)\right)+\sum_{j=1}^{k}\sum_{i=1}^{n_{j}}\frac{2i-1}{n_{j}}\frac{1}{F\left(x_{ij};a,b,\beta \right)}.$$

The estimators of a,b and β are obtained by solving the corresponding non-linear equations given in Appendix H. The ADLTS estimators of $\alpha_{j},\;j=1,\ldots ,k$ are obtained as follows:

$${\hat{\alpha }}_{ADLTSj}=e^{{\hat{a}}_{ADLTS}+{\hat{b}}_{ADLTS}S_{j}}.$$

### 3.9. Method of Maximum Product of Spacing

The MPS method, introduced by Cheng and Amin [39] was proposed as an alternative to the ML estimation for continuous univariate distributions. This nonparametric approach estimates the model parameters by maximizing the product of the observed spacings between the ordered data points:

(6) $$D_{ij}\left(a,b,\beta \right)=F\left(x_{ij}|a,b,\beta \right)-F\left(x_{i-1j}|a,b,\beta \right).$$

The MPS estimators of unknown parameters, say ${\hat{a}}_{MPS},\;{\hat{b}}_{MPS}$ and ${\hat{\beta }}_{MPS}$, respectively, are derived by maximizing the following function:

$$M\left(a,b,\beta \right)=\sum_{j=1}^{k}\sum_{i=1}^{n_{j}+1}\frac{lnD_{ij}(a,b,\beta )}{n_{j}+1}.$$

The estimators of a,b and β are obtained by solving the corresponding non-linear equations given in Appendix I. The MPS estimators of $\alpha_{j},\;j=1,\ldots ,k$ are obtained as follows:

$${\hat{\alpha }}_{MPSj}=e^{{\hat{a}}_{MPS}+{\hat{b}}_{MPS}S_{j}}.$$

### 3.10. Method of Minimum Spacing Absolute Distance

The MSAD estimators of a,b and β, namely ${\hat{a}}_{MSAD},\;{\hat{b}}_{MSAD}$ and ${\hat{\beta }}_{MSAD}$, are derived by minimizing the following function:

(7) $$T\left(a,b,\beta \right)=\sum_{j=1}^{k}\sum_{i=1}^{n_{j}+1}\left|D_{ij}\left(a,b,\beta \right)-\frac{1}{n_{j}+1}\right|.$$

The estimators of the unknown parameters are obtained by solving the corresponding non-linear equations given in Appendix J. The MSAD estimators of $\alpha_{j},\;j=1,\ldots ,k$ are obtained as follows:

$${\hat{\alpha }}_{MSADj}=e^{{\hat{a}}_{MSAD}+{\hat{b}}_{MSAD}S_{j}}.$$

### 3.11. Method of Minimum Spacing Absolute-Log Distance

The MSALD estimators of the unknown parameters a,b and β, namely ${\hat{a}}_{MSALD},\;{\hat{b}}_{MSALD}$ and ${\hat{\beta }}_{MSALD}$, are derived by minimizing the following function:

(8) $$MS\left(a,b,\beta \right)=\sum_{j=1}^{k}\sum_{i=1}^{n_{j}+1}\left|\ln D_{ij}\left(a,b,\beta \right)-\ln\frac{1}{n_{j}+1}\right|.$$

The estimators of a,b and β are obtained by solving the corresponding non-linear equations given in Appendix K. The MSALD estimators of $\alpha_{j},\;j=1,\ldots ,k$ are obtained as follows:

$${\hat{\alpha }}_{MSALDj}=e^{{\hat{a}}_{MSALD}+{\hat{b}}_{MSALD}S_{j}}.$$

## 4. Simulation Study

In this section, a Monte Carlo simulation study is conducted to compare the performance of the different estimators discussed in Section 3. The data are generated under the CSALT model by fixing the scale parameter at β=2 and considering various combinations of the stress–life relationship parameters $\left(a,b\right)$.

The combinations, $\left(a,b\right)=\left(0.2,\;0.2\right),\;\left(0.2,\;0.5\right),\;\left(0.2,\;0.8\right),\;\left(0.5,\;0.2\right),\;\left(0.5,\;0.8\right),\;(0.8,\;0.8)$ are deliberately selected to represent a wide range of practical scenarios. In particular, these combinations allow us to capture:

- low, moderate, and high baseline effects through parameter a,
- weak to strong stress sensitivity through parameter b,
- and different growth rates in the shape parameter $\alpha_{j}=\exp\left(a+bS_{j}\right)$.

Two stress levels ($S_{1}=0.2$ and $S_{2}=0.4$) are considered in CSALT setting. The selected sample sizes N=(30, 60, 120) allow us to evaluate estimator performance under small, moderate, and large sample regimes.

In the primary design, samples are equally allocated between stress levels (i.e., $n_{j}=N/2,\;j=1,2$), which is consistent with standard practice in the CSALT literature, where balanced designs are preferred for ensuring comparability and statistical stability. Additional simulations are also conducted to examine the effect of unequal sample allocation. First, the original two-stress-level setting is reconsidered using unequal sample allocations across the stress levels. Second, the simulation design is extended to three accelerated stress levels with unequal sample allocations. The results show that the general performance patterns of the estimation methods remain largely consistent with those obtained under the two-level balanced design considered in the main simulation study. Although some differences in bias and mean square error (MSE) are observed across the alternative designs, the main comparative conclusions regarding the estimation methods are not substantially altered. Therefore, to maintain clarity and avoid substantially increasing the number of tables in the main text, the two-level balanced design is retained as the primary simulation setting. The complete results for the two-level unbalanced and three-level unbalanced designs are provided in Tables S4–S6 of the Supplementary Materials.

For each combination of parameters, the bias and the corresponding MSE values are computed in order to compare the performance of the proposed estimators:

$$Bias=\sum_{t=1}^{1000}\frac{{\hat{\theta }}_{t}}{t}-\theta \;\mathrm{and}\;MSE=\sum_{t=1}^{1000}\frac{{\left({\hat{\theta }}_{t}-\theta \right)}^{2}}{t},\theta =a,b,\beta ,\alpha_{1},\alpha_{2}.$$

Accordingly, the most efficient estimation method is defined as the one whose MSE values are closest to zero. All computations are carried out in Matlab R2024b with 1000 replications using “*fminunc*” function. For each point estimation method, the corresponding objective functions are constructed and minimized or maximized. The optimization is performed using appropriate initial values as starting points, with a maximum of 5000 iterations to ensure convergence. The results are presented in Table 1, Table 2 and Table 3.

The shape parameter $\alpha_{u}$ under the normal-use condition $S_{u}$ is estimated using the following equation:

$${\hat{\alpha }}_{u}=e^{{\hat{a}}^{*}+{\hat{b}}^{*}S_{u}},$$

Here, ${\hat{a}}^{*}$ and ${\hat{b}}^{*}$ denote the parameter estimates obtained using different estimation methods. Similarly, under the normal-use condition $S_{u}$, the estimated value of the reliability function at the lifetime $x_{0}$ is computed through the following equation:

$${\hat{R}}_{u}(x_{0})=1-{\left(\frac{1-e^{-{\hat{\beta }}^{*}x_{0}}}{1+e^{-{\hat{\beta }}^{*}x_{0}}}\right)}^{{\hat{\alpha }}_{u}},$$

where ${\hat{\beta }}^{*}$ is the estimate of β obtained by the different methods. For different values of N, the estimated values of the shape parameter and the reliability function are obtained. The normal-use stress level is set to $S_{u}=0.1$, which is lower than the accelerated stress levels $S_{1}=0.2$ and $S_{2}=0.4$. This value is selected to represent a lower-stress normal operating condition and to evaluate the reliability and MTTF by extrapolating the fitted stress–life relationship from the accelerated stress levels to the normal-use condition. The estimated shape parameter values are presented in Table 4, while the estimated values of the reliability function for some selected mission times are reported in Table 5, Table 6 and Table 7.

The simulation results indicate that the performance of the estimators depends on the sample size and parameter settings, see Table 1, Table 2 and Table 3. In terms of biases of the estimators, the MPS, MSAD, and MSALD estimators consistently exhibit negative bias across all sample sizes. This behavior suggests a systematic tendency of these methods to slightly underestimate the parameters, which may be attributed to their sensitivity to the tail and shape characteristics of the underlying distribution. In contrast, the LS and WLS methods display negative bias only in certain scenarios, indicating a higher dependence on the specific data configuration. Although the magnitude of bias generally decreases as the sample size increases, as expected.

In view of MSEs, for small and moderate sample sizes, the MPS estimator yields lower MSE values, which can be attributed to its more efficient use of the cumulative information structure, leading to better variance control in limited samples. The improved performance of the ML and AD methods as the sample size increases is consistent with their asymptotic efficiency and reduced variance in large samples. In contrast, the relatively poorer performance of the CM, RTAD, and ADLTS methods can be explained by their higher sensitivity to data fluctuations and their tendency to produce larger variances. Overall, the results show that the MPS method is more stable and efficient in small and moderate samples, while the ML and AD methods become more competitive in large-sample settings due to their asymptotic properties.

To assess the effect of violating the common β assumption, an additional sensitivity analysis is conducted. The data are generated by allowing the scale parameter to vary across stress levels according to $\beta_{j}=exp(c+dS_{j})$, where β=2, $S_{u}=0.1$, d=0.5, and $c=\log\left(\beta \right)-dS_{u}$. Accordingly, $\beta_{1}=2.1025$ and $\beta_{2}=2.2103$ are obtained for $S_{1}=0.2$ and $S_{2}=0.4$, respectively, while estimation is still performed under the common β model considered in the main simulation study. The results show that this model misspecification affects estimation accuracy to some extent; however, the main comparative findings among the estimation methods remain largely unchanged. In particular, MPS and MSALD continue to exhibit relatively stable performance. These findings suggest that the main conclusions are reasonably stable under the considered departure from the common β assumption, although ignoring stress-related variation in β may introduce additional estimation error. Detailed results are provided in Tables S1–S3 of the Supplementary Materials.

To provide a clearer comparison of the estimation methods across the considered simulation settings, the best-performing methods in terms of bias and MSE are also identified for each sample size and parameter configuration. The results show that the relative performance of the estimators depends on both the sample size and the parameter setting. In terms of MSE, MPS generally exhibits strong performance, particularly for small and moderate sample sizes, whereas ML becomes more competitive as the sample size increases and provides the best performance in some large-sample settings. The method yielding the smallest bias varies more noticeably across the parameter configurations, indicating that no single estimator uniformly minimizes bias. Overall, the effect of sample size on the relative performance is more pronounced than that of the parameter configuration. A detailed summary of the best-performing methods for each simulation setting is provided in Tables S7 and S8 of the Supplementary Materials.

Table 4 presents the estimated values of the shape parameter for different values of a and b under $S_{u}=0.1$. It is observed from this table that, for all estimation methods, the estimates of the shape parameter become more stable as the sample size increases, and the differences among the methods decrease substantially. With respect to different values of a and b, it is observed that the estimated values of the shape parameter increase across all methods as the parameters a and b increase. This finding is consistent with the structure of the model and indicates that the shape parameter is sensitive to the stress and distribution parameters.

From Table 5, Table 6 and Table 7, it is observed that, for all estimation methods, the estimated values of the reliability function decrease in a regular and pronounced manner as the mission time $x_{0}$ increases. When examined across different combinations of *a* and *b*, the estimated reliability values generally increase with increasing values of the parameters *a* and *b*. Furthermore, it is noted that these estimated values are not significantly affected by increases in the sample size.

**Figure 2:**
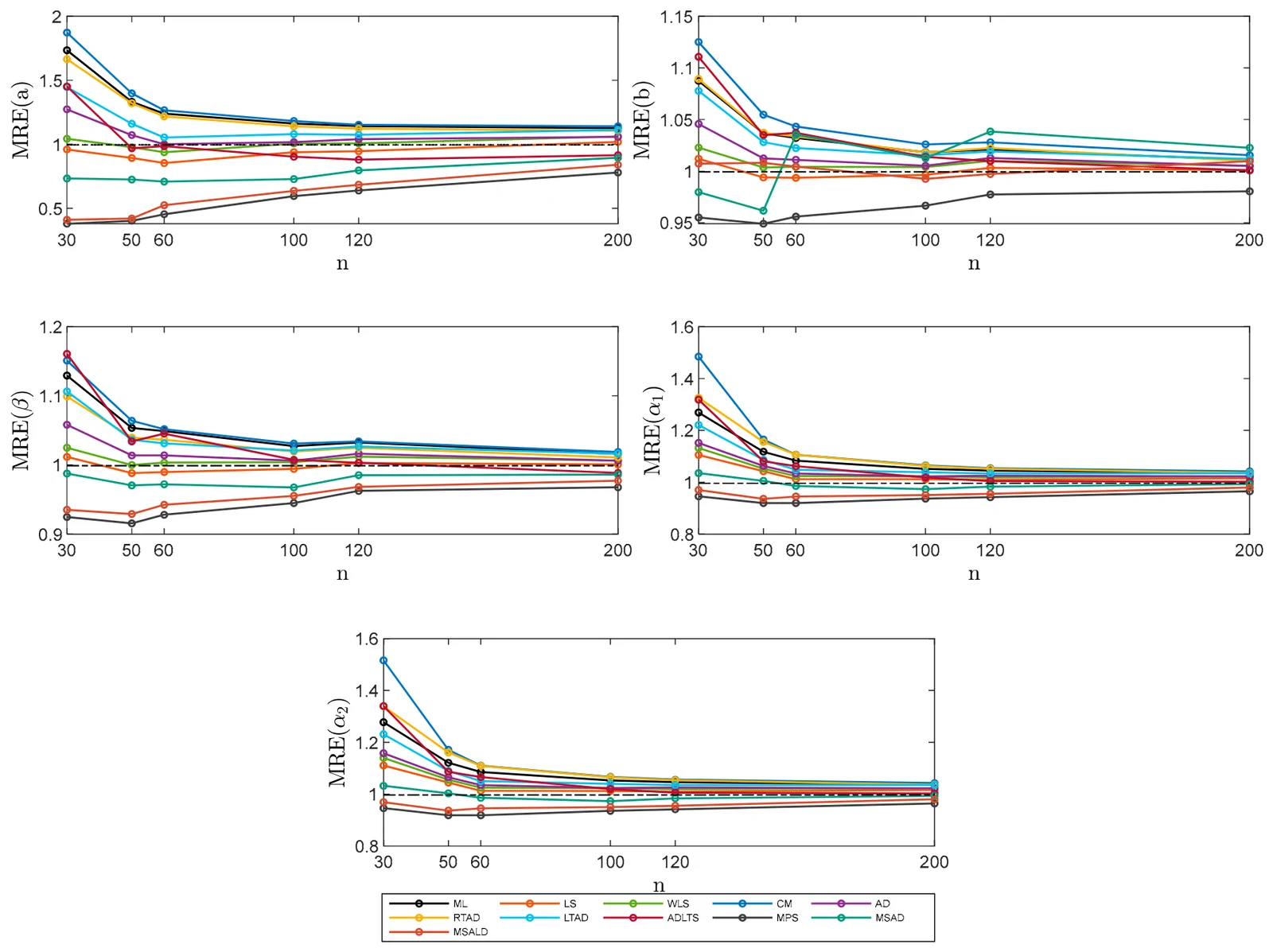
MREs of various estimates of $a,b,\beta ,\alpha_{1},$ and $\alpha_{2}$ when a=b=0.2.

**Figure 3:**
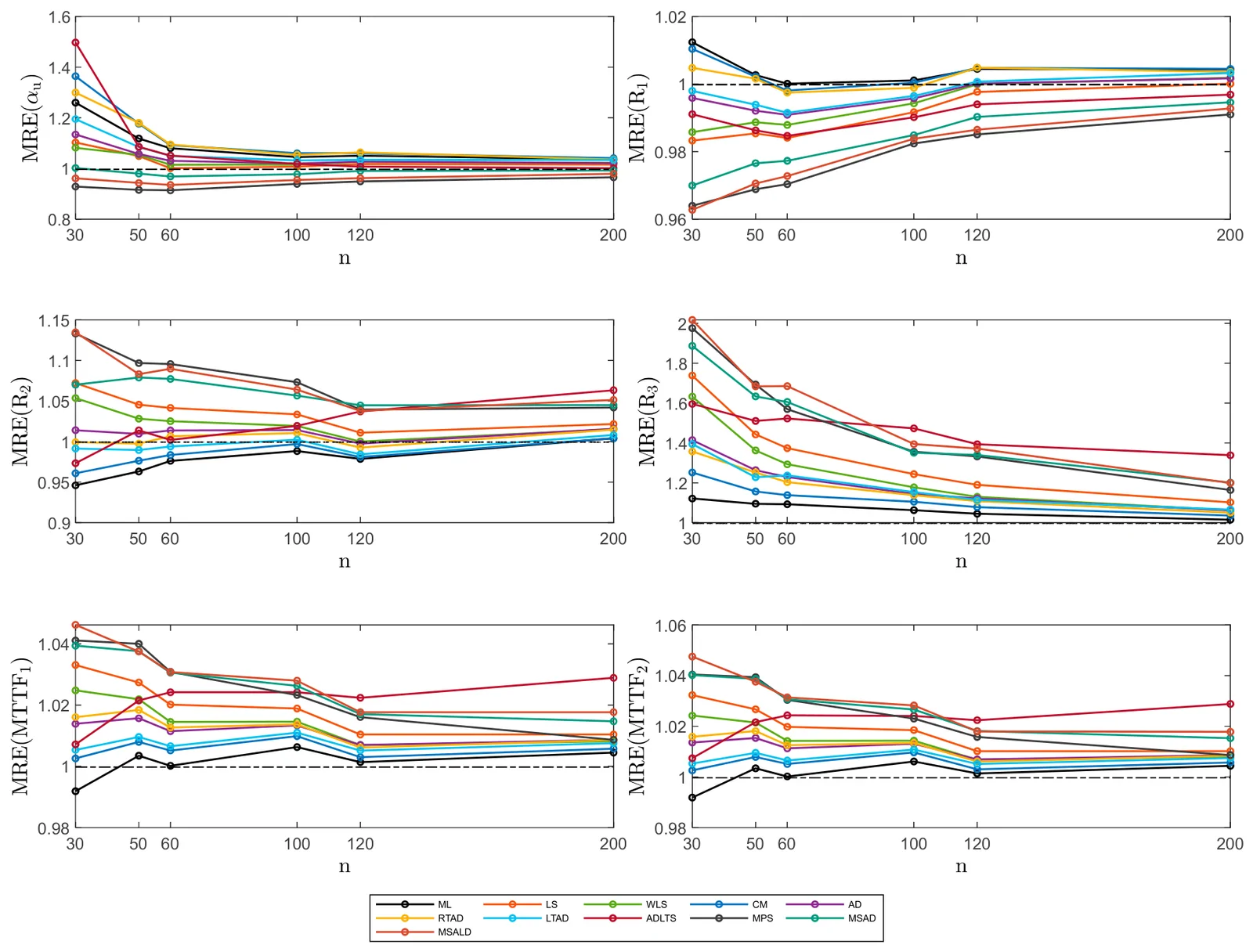
MREs of various estimates of $\alpha_{u},R\left(x\right),$ and MTTF when a=b=0.2.

**Figure 4:**
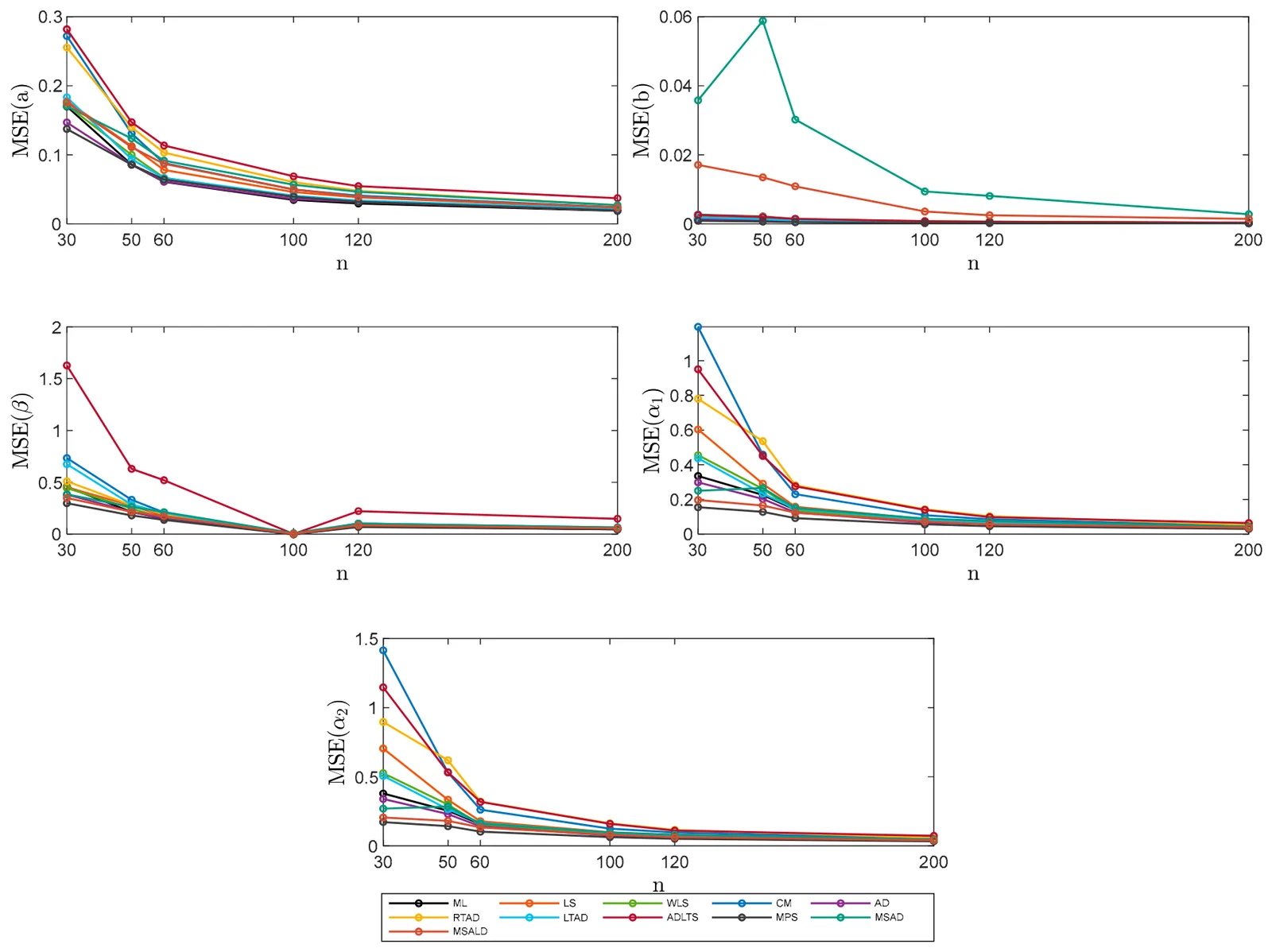
MSEs of various estimates of $a,b,\beta ,\alpha_{1},$ and $\alpha_{2}$ when a=b=0.2.

**Figure 5:**
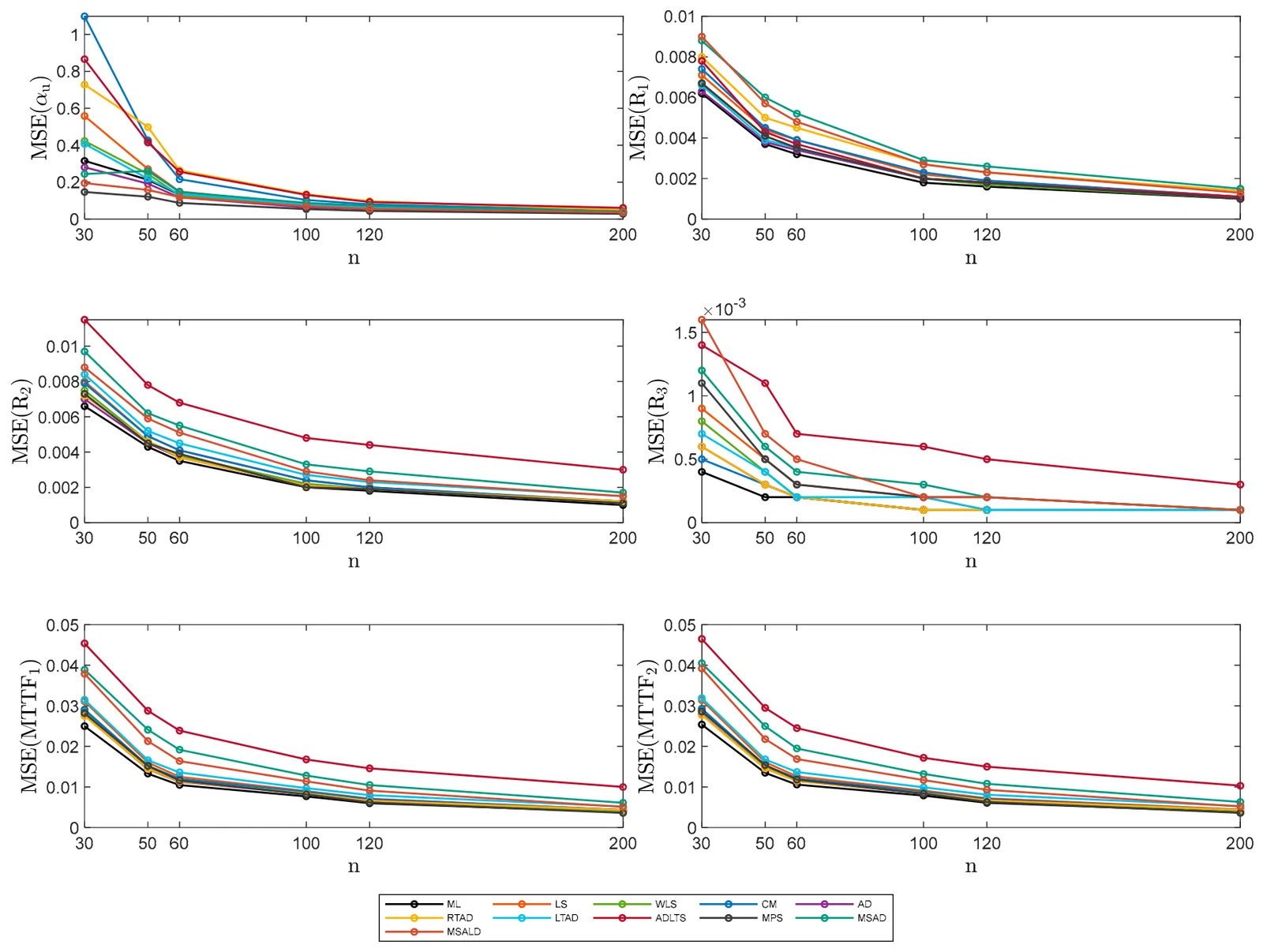
MSEs of various estimates of $\alpha_{u},R\left(x\right),$ and MTTF when a=b=0.2.

**Figure 6:**
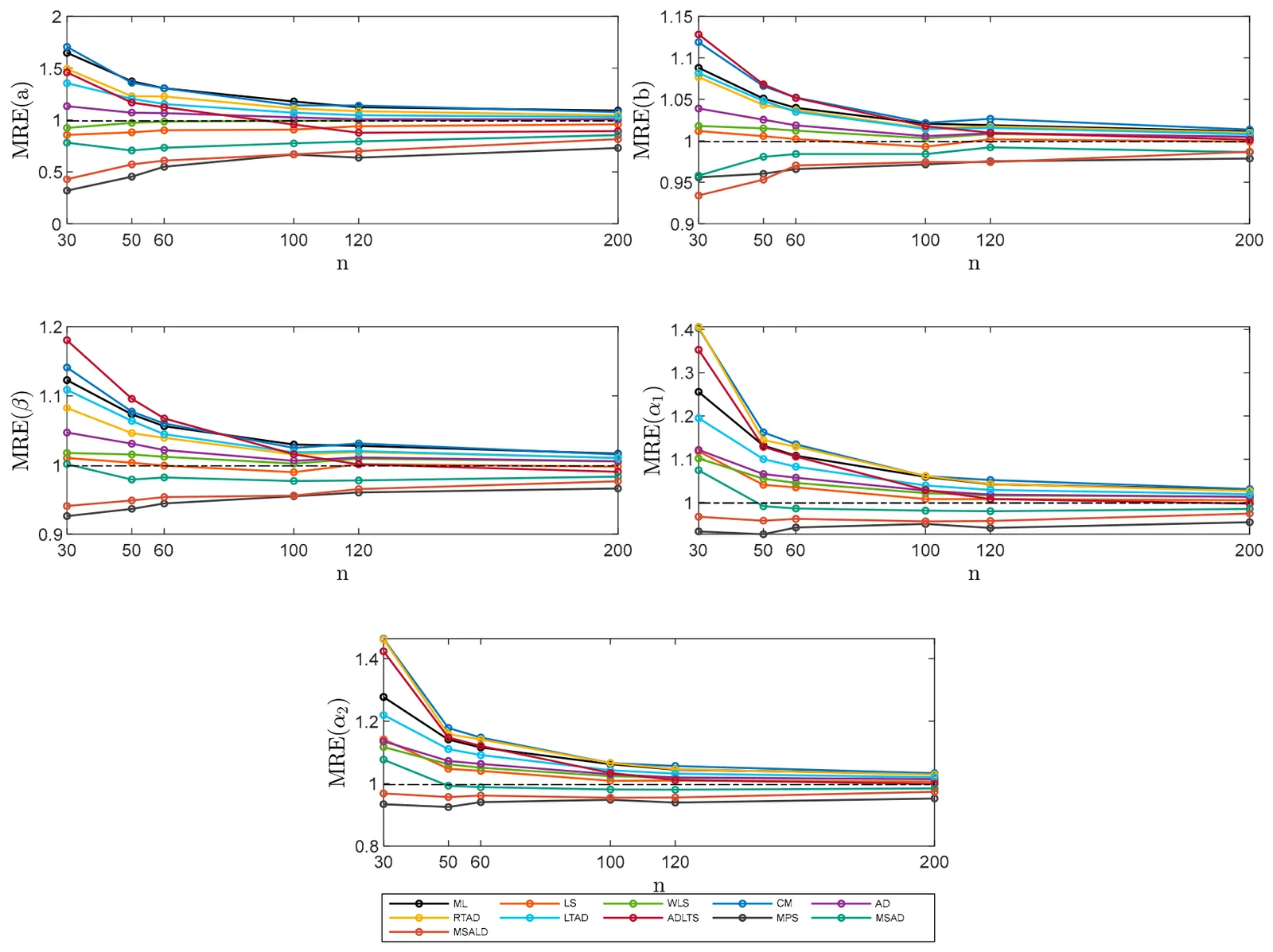
MREs of various estimates of $a,b,\beta ,\alpha_{1},$ and $\alpha_{2}$ when a=0.2, b=0.5.

**Figure 7:**
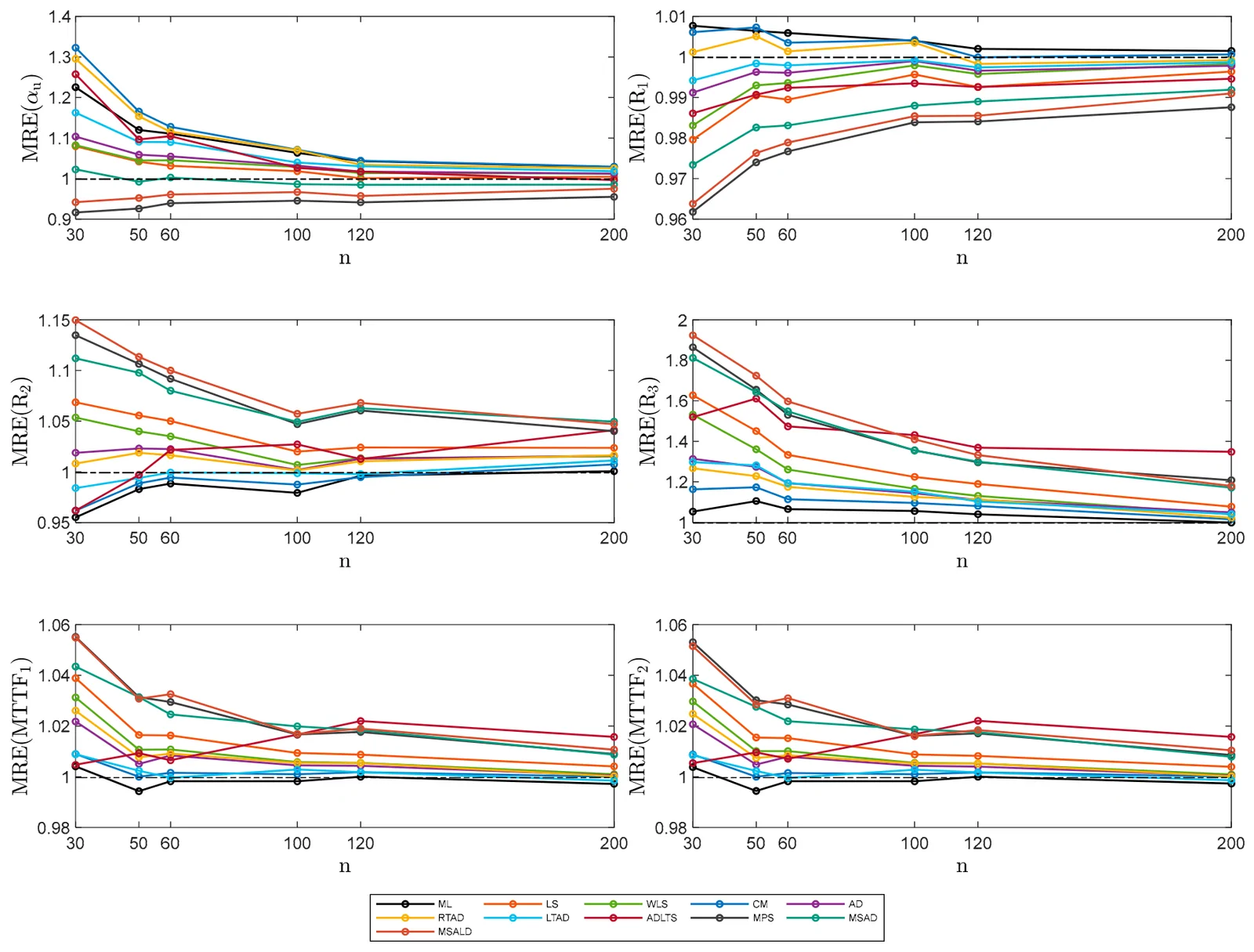
MREs of various estimates of $\alpha_{u},R\left(x\right),$ and MTTF when a=0.2, b=0.5.

**Figure 8:**
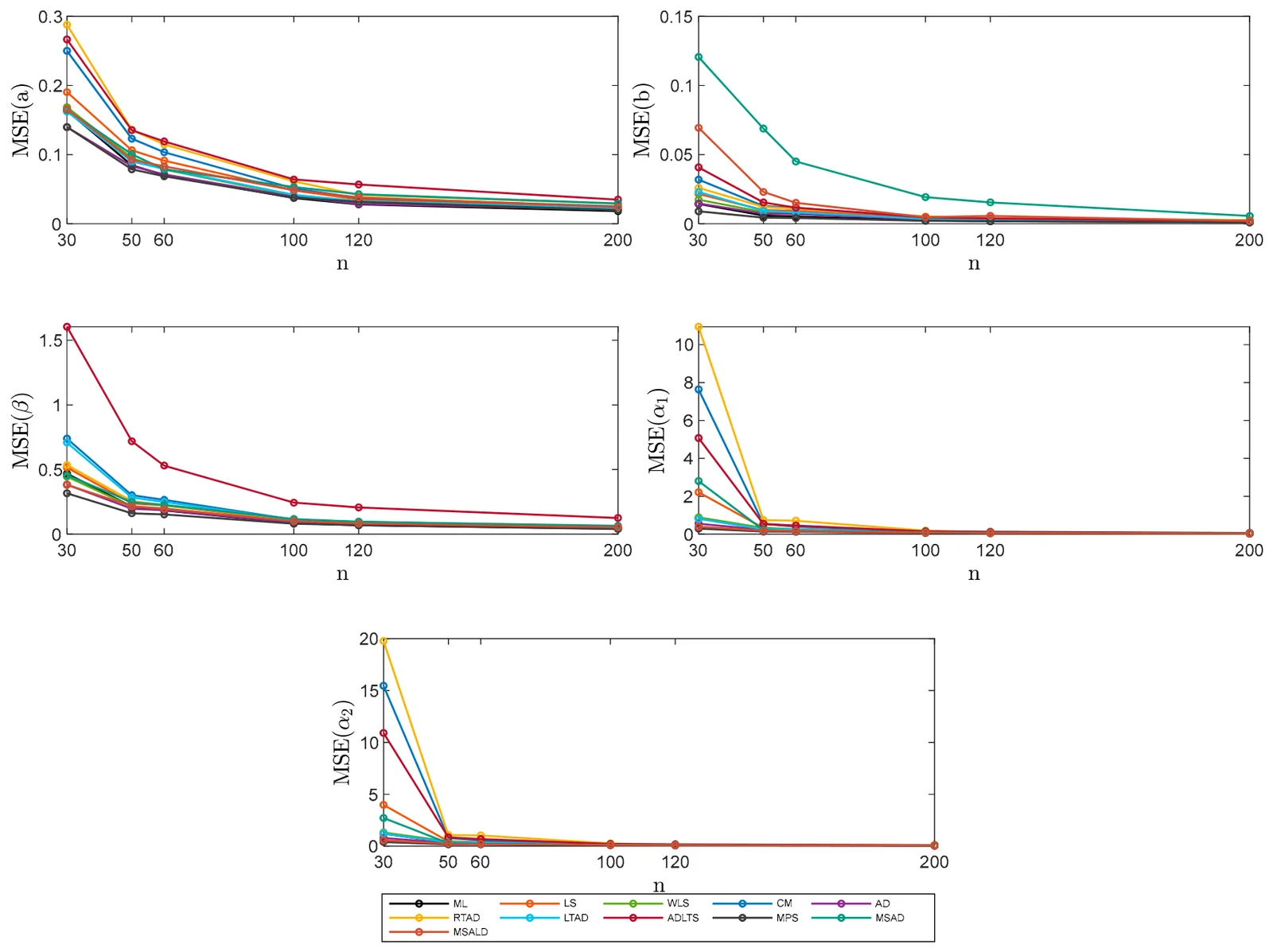
MSEs of various estimates of $a,b,\beta ,\alpha_{1},$ and $\alpha_{2}$ when a=0.2, b=0.5.

**Figure 9:**
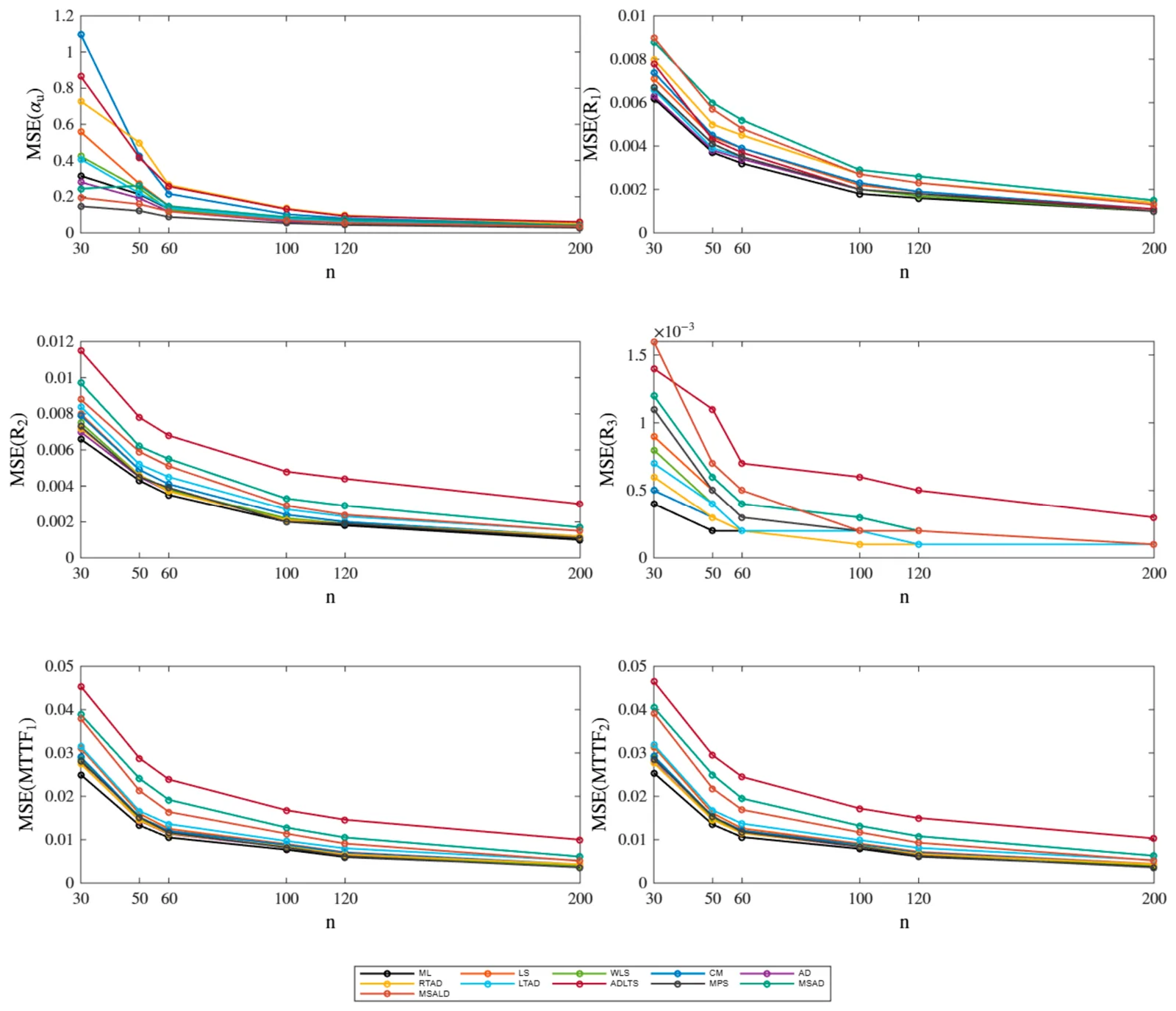
MSEs of various estimates of $\alpha_{u},R\left(x\right),$ and MTTF when a=0.2, b=0.5.

The average values of the mean relative estimates (MREs) and MSEs of model parameters, say $a,\;b,\;\beta ,\;\alpha_{1},\;\alpha_{2},\;\alpha_{u}$, and also $R\left(x\right)$ and MTTF functions are computed and presented in Figure 2, Figure 3, Figure 4, Figure 5, Figure 6, Figure 7, Figure 8 and Figure 9. Figure 2, Figure 3, Figure 4 and Figure 5 display the MREs and MSEs for the case (a,b)=(0.2, 0.2), whereas Figure 6, Figure 7, Figure 8 and Figure 9 correspond to the parameter combination (a,b)=(0.2, 0.5).

The MRE figures clearly indicate that, for all estimation methods, the MRE values converge to 1 as the sample size increases. This finding demonstrates that all the considered estimators are asymptotically unbiased. For large sample sizes, i.e., N≥100, the differences among the estimation methods become negligible, and all estimators exhibit comparable performance. This observation highlights that increasing the sample size substantially enhances estimation accuracy.

The MSE plots further reveal that, for all estimation methods, the MSE values decrease rapidly and in a regular manner as the sample size increases. This result provides clear evidence of the consistency of the estimators. Overall, the combined MRE and MSE results indicate that all the proposed methods yield asymptotically unbiased and consistent estimators for the EHL distribution under the CSALT framework.

## 5. Real Data Analysis

In this section, a real-life dataset obtained from the Elsayed [40] is analyzed. The dataset consists of the times to failure of 6H-SiC MOS capacitors subjected to different temperature conditions. For computational convenience, the failure times originally recorded in hours are scaled by dividing each observation by 1000, and all analyses are conducted using the scaled data. The dataset for three different stress levels at 145 °C, 240 °C and 305 °C are presented in Table 8. It should be stated that the observed temperature levels are directly used as the stress covariate $S_{j}$ in the relationship $\alpha_{j}=exp(a+bS_{j})$. Thus, the model assumes a linear relationship between $log(\alpha_{j})$ and the stress covariate, rather than between $\alpha_{j}$ and temperature itself. The direct use of temperature is adopted here to apply the stress–shape relationship considered in the proposed CSALT model, with the primary aim of comparing the estimation methods rather than developing a physically based temperature-acceleration model. We acknowledge that an Arrhenius-type transformation may provide a more physically motivated representation when temperature is the accelerating variable. Investigating alternative stress transformations is therefore considered a direction for future research.

**Figure 10:**
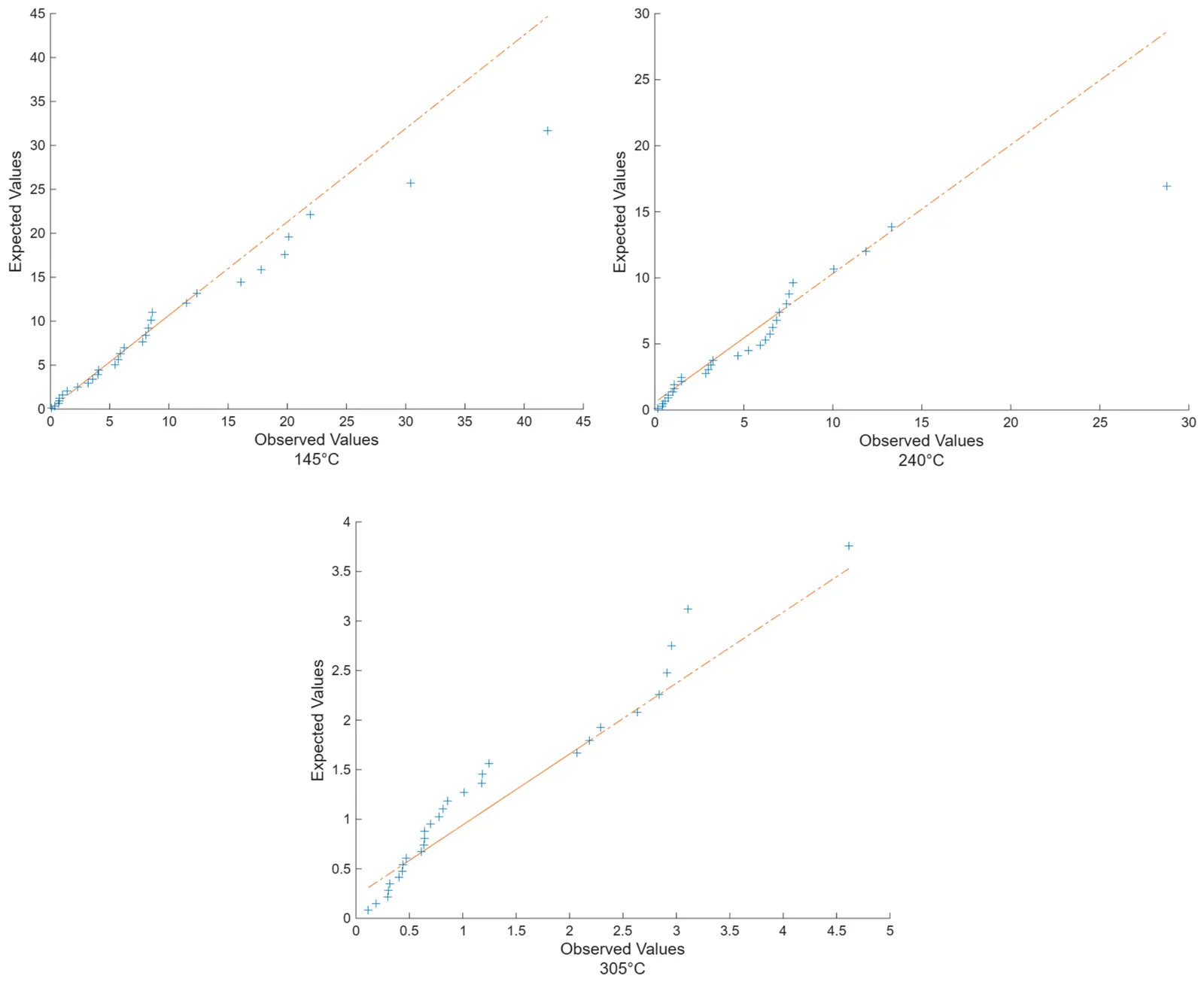
The EHL Q–Q plots for different stress levels.

To verify the adequacy of the EHL distribution for modeling the dataset, goodness-of-fit tests are conducted, and its performance is compared with several well-established alternative distributions, including the Generalized Logistic (GL), Exponentiated Rayleigh (ER), Generalized Inverse Lindley (GIL), and Exponentiated Lindley (EL) distributions, as shown in Table 9. These alternative distributions are specifically selected because they have been previously used as underlying lifetime models in CSALT studies, thereby providing a meaningful and literature-supported basis for comparison. For the goodness-of-fit comparison, the parameters of all candidate distributions are estimated using ML to provide a common and consistent basis for distribution selection. The purpose of this stage is to compare the suitability of the candidate lifetime distributions rather than to investigate the effect of different estimation methods on distributional fit. After establishing the suitability of the EHL distribution based on this comparison, the subsequent analysis focuses on comparing the eleven estimation methods within the EHL-based CSALT model. In this context, the Kolmogorov–Smirnov (KS) statistic and its associated *p*-value, along with the root mean squared error (RMSE), coefficient of determination ($R^{2}$), Akaike information criterion (AIC), and Bayesian information criterion (BIC) are computed. In addition, Q–Q plots of the EHL distribution are presented for each dataset in Figure 10. The obtained results indicate that the EHL distribution provides a satisfactory and stable fit to the considered datasets.

To estimate the CSALT model parameters based on EHL distribution, we use the estimation methods given in Section 3. The resulting parameter estimates are reported in Table 10. In addition, based on 1000 bootstrap samples, the estimated standard errors (ESEs) corresponding to each estimation method are computed and also presented in Table 10. Table 10 indicates that for the parameters a, b, and β, the MSAD and LTAD methods yield comparatively smaller ESE values than the other methods. With respect to the shape parameters, the RTAD, LS, and LTAD methods exhibit relatively better performance. Moreover, the MPS method consistently produces low ESE values, thereby confirming its efficiency as an estimation technique in the context of the real data analysis.

The estimated MTTF values under different stress levels, along with their corresponding ESEs, are presented in Table 11. From this table, the ML and WLS methods generally exhibit the lowest ESEs, demonstrating superior performance compared with the other estimation techniques.

Table 12 demonstrates the estimates of the reliability function at selected time points and the corresponding shape parameter, together with their ESEs, are reported under the usual stress level. Here, $S_{u}={125\;}^{\circ }C$ is selected as a representative stress level below the lowest accelerated test temperature of ${145\;}^{\circ }C$. This value is used as an illustrative normal-use condition rather than a value specified in the original data source. Overall, the estimates of R(x) are observed to decrease as the values of $x_{0}$ increase, which is consistent with the theoretical expectation that system reliability deteriorates over time. In terms of estimation precision, ML and MSAD methods yield the smallest ESE values for smaller $x_{0}$ values, whereas for larger $x_{0}$ values, the MPS and ML methods provide comparatively lower ESEs than the remaining approaches. Moreover, in terms of the estimates of the shape parameter, the MSALD and AD methods stand out by producing the lowest ESE, indicating a relatively superior performance in estimating this parameter.

Table 13 represents the comparison of the percentile bootstrap confidence interval lengths obtained using different estimation methods. Shorter confidence interval lengths indicate higher estimation precision and lower associated uncertainty. From a general perspective, the MPS and MSALD methods yield shorter confidence intervals for most parameters and reliability measures. In contrast, the WLS and ADLTS methods tend to produce wider confidence intervals across several parameters. Focusing specifically on the MTTF estimates, the MPS and WLS methods generate shorter confidence intervals, whereas the ADLTS method produces the widest intervals across all stress levels. This finding suggests that the MPS method demonstrates strong performance not only in estimating the model parameters but also in the estimation of derived reliability measures. It can be concluded from Table 13 that the MPS and MSALD methods generally provide higher estimation precision, while the ADLTS and WLS methods exhibit relatively weaker performance for certain parameters and reliability measures.

## 6. Conclusions

In this study, eleven estimation methods, namely ML, LS, WLS, CM, AD, RTAD, LTAD, ADLTS, MPS, MSAD, and MSALD, are considered for estimating the parameters, reliability function, and MTTF of the EHL-based CSALT model. Their performances are investigated through an extensive Monte Carlo simulation study under different sample sizes and parameter settings and are further examined using a real accelerated life-testing dataset.

The simulation results reveal clear differences among the estimation methods. In particular, MPS provides strong overall performance in terms of MSE especially for small and moderate sample sizes. MSALD also performs competitively in many cases. The performance of ML improves noticeably as the sample size increases; while its performance is more moderate for small samples, it achieves the second overall rank for all parameter settings for large sample size. AD also provides competitive results, particularly for small and moderate sample sizes. These findings indicate that the relative performance of the estimation methods is affected more noticeably by sample size than by the considered values of the model parameters. However, CM, RTAD, and ADLTS generally produce relatively large MSE values under the simulation settings considered in this study and should therefore be used with caution. These recommendations are specific to the conditions investigated here and should not be interpreted as general rankings of the estimation methods.

The real data analysis generally supports the simulation findings and demonstrates the practical applicability of the EHL-based CSALT model. MPS yields comparatively low ESE values and produces the shortest bootstrap confidence intervals among the competing methods. The analysis also shows that the estimated MTTF decreases as the stress level increases for all estimation methods. Overall, the results show that alternative estimation methods, particularly MPS, can provide useful options in addition to the conventional ML method for reliability and lifetime estimation under accelerated testing conditions.

One limitation of the present study is that the analysis is restricted to complete failure data. Since censoring is frequently encountered in practical ALT studies, future research may extend the proposed model by considering different censoring schemes. Bayesian estimation methods may also be investigated as alternatives to the classical estimation methods considered in this study. Another interesting direction is to examine the performance of the EHL distribution under different ALT models, such as step-stress accelerated life testing (SSALT), constant-stress partially accelerated life testing (CSPALT), and step-stress partially accelerated life testing (SSPALT). In addition, a systematic investigation of the coverage probabilities and interval widths of different confidence interval procedures may provide a useful extension of the present point-estimation-focused simulation study.

## Figures and Tables

**Table 1:** The biases (first row) and the MSEs (second row) of the estimators for N=30.

*a*	*b*	Par	ML	LS	WLS	CM	AD	RTAD	LTAD	ADLTS	MPS	MSAD	MSALD
0.2	0.2	a	0.1469	−0.008	0.0087	0.1745	0.0546	0.1329	0.0892	0.0903	−0.1241	−0.0534	−0.118
			0.1701	0.1778	0.1719	0.2719	0.1469	0.2554	0.1833	0.2817	0.1375	0.1705	0.1755
		b	0.0175	0.0024	0.0046	0.025	0.0092	0.0178	0.0156	0.0221	−0.0090	−0.0040	0.0015
			0.0020	0.0029	0.0027	0.0049	0.0022	0.0038	0.0034	0.0058	0.0013	0.0584	0.0373
		β	0.2588	0.0234	0.05	0.302	0.1164	0.1983	0.2126	0.3213	−0.1504	−0.0249	−0.1296
			0.4578	0.4539	0.4447	0.7339	0.3822	0.5100	0.6746	1.6279	0.2989	0.3844	0.3487
		$\alpha_{1}$	0.3423	0.1341	0.1686	0.616	0.1931	0.4149	0.281	0.4052	−0.0689	0.0447	−0.0378
			0.8534	0.7736	1.5211	20.583	0.6353	1.6267	0.994	2.8066	0.2957	0.4412	0.3919
		$\alpha_{2}$	0.3674	0.1468	0.1854	0.6828	0.2089	0.4485	0.3058	0.4499	−0.0713	0.0428	−0.0402
			0.9876	0.8972	1.8777	28.5688	0.7404	1.8983	1.1788	3.6115	0.3344	0.4596	0.4163
0.2	0.5	a	0.1296	−0.0284	−0.0151	0.141	0.0268	0.0989	0.0711	0.0917	−0.136	−0.0436	−0.1142
			0.1637	0.1904	0.1686	0.2501	0.1398	0.2878	0.1623	0.2666	0.1397	0.1648	0.1657
		b	0.044	0.0059	0.0089	0.0595	0.0194	0.0385	0.0408	0.064	−0.022	−0.021	−0.033
			0.0143	0.0216	0.0174	0.0319	0.0146	0.026	0.023	0.0408	0.0090	0.1206	0.0694
		β	0.2456	0.0204	0.0352	0.2821	0.0939	0.1652	0.2177	0.3613	−0.1474	0.0029	−0.1184
			0.4684	0.5151	0.4458	0.7396	0.3836	0.5348	0.7088	1.6044	0.3168	0.4561	0.3817
		$\alpha_{1}$	0.3452	0.1596	0.1373	0.5445	0.164	0.5486	0.2632	0.4766	−0.0904	0.1007	−0.0444
			0.8426	2.2141	0.8895	7.6413	0.5561	10.9454	0.8069	5.0743	0.3010	2.8056	0.4000
		$\alpha_{2}$	0.4142	0.2119	0.1749	0.6926	0.2018	0.6891	0.3279	0.6324	−0.0978	0.115	−0.0466
			1.2058	3.986	1.3131	15.4606	0.7913	19.7794	1.2102	10.9114	0.4083	2.7137	0.5573
0.2	0.8	a	0.1202	−0.027	−0.0186	0.1432	0.0227	0.0907	0.0685	0.0825	−0.1445	−0.0559	−0.1317
			0.1633	0.2078	0.1891	0.2808	0.1445	0.258	0.1875	0.2966	0.1460	0.1551	0.1589
		b	0.0659	0.0081	0.0104	0.0955	0.027	0.058	0.0624	0.0982	−0.0453	−0.0405	−0.0481
			0.0364	0.0548	0.0466	0.0825	0.0361	0.069	0.0627	0.1192	0.0237	0.1691	0.0727
		β	0.2206	0.0117	0.0174	0.2709	0.0765	0.1522	0.1928	0.3141	−0.1792	−0.0404	−0.154
			0.4260	0.4756	0.4234	0.6908	0.3475	0.5322	0.6442	1.4798	0.3021	0.4252	0.3586
		$\alpha_{1}$	0.3754	0.2838	0.2459	0.8555	0.1895	0.5161	0.3993	0.9274	−0.1037	0.0556	−0.0715
			1.2214	15.3799	8.3889	69.652	0.9374	6.4165	8.718	77.5935	0.4082	0.7678	0.4914
		$\alpha_{2}$	0.504	0.4368	0.3701	1.2829	0.2634	0.7379	0.6283	1.7427	−0.1192	0.0906	−0.0747
			2.2961	37.7563	19.4747	192.8995	1.7373	14.5786	36.8974	381.9324	0.6842	1.4203	0.8526
0.5	0.2	a	0.1542	−0.0131	−0.0028	0.185	0.0493	0.142	0.0868	0.1027	−0.1482	−0.068	−0.1322
			0.1863	0.2435	0.2126	0.346	0.1711	0.3624	0.2007	0.2904	0.1487	0.1783	0.1825
		b	0.0177	0.0024	0.0036	0.0241	0.0083	0.0169	0.0143	0.0219	−0.0101	0.0139	0.008
			0.0021	0.0032	0.0027	0.0048	0.0022	0.0041	0.003	0.005	0.0013	0.0099	0.0077
		β	0.2285	0.0154	0.0279	0.2654	0.0916	0.1759	0.1689	0.2726	−0.1537	−0.0395	−0.1255
			0.3673	0.4229	0.3753	0.6106	0.3189	0.4915	0.4715	1.0244	0.2548	0.3377	0.2996
		$\alpha_{1}$	0.4826	0.2835	0.2484	0.9261	0.2718	1.0434	0.3863	0.5712	−0.1345	0.0508	−0.0762
			1.3291	3.6353	2.3423	16.691	1.122	72.6046	1.7466	4.8761	0.4245	0.8032	0.5465
		$\alpha_{2}$	0.5173	0.3121	0.272	1.0095	0.2937	1.1403	0.419	0.6296	−0.14	0.0596	−0.0779
			1.5202	4.3367	2.7509	20.2771	1.2892	89.5631	2.0561	6.1102	0.4751	0.9031	0.5895
0.5	0.8	a	0.1393	−0.0249	−0.018	0.1681	0.031	0.1159	0.0717	0.0974	−0.1529	−0.083	−0.143
			0.1901	0.2287	0.1906	0.3176	0.1624	0.312	0.1975	0.3095	0.1637	0.1890	0.1885
		b	0.0708	0.0073	0.0106	0.0951	0.0283	0.061	0.0562	0.096	−0.045	−0.009	−0.0285
			0.0400	0.0535	0.0452	0.0805	0.0382	0.0668	0.0532	0.0993	0.0261	0.0795	0.0466
		β	0.2123	0.0088	0.0178	0.2545	0.0746	0.1556	0.1574	0.2718	−0.1624	−0.0673	−0.1413
			0.3807	0.4191	0.3687	0.6096	0.3186	0.4782	0.4737	1.027	0.2697	0.3377	0.3007
		$\alpha_{1}$	0.6324	0.3322	0.2460	1.0836	0.3233	0.9789	0.5088	0.9208	−0.1189	0.0808	−0.0666
			6.7105	7.1966	2.9755	29.9413	3.8841	54.2534	7.9837	22.1792	1.5977	3.5056	1.9686
		$\alpha_{2}$	0.8603	0.4875	0.3582	1.5347	0.4535	1.4396	0.7212	1.4149	−0.125	0.1374	−0.0533
			14.2507	14.4197	5.7389	66.4687	7.9700	164.5371	17.6223	61.1241	2.9476	7.2877	3.7438
0.8	0.8	a	0.1898	−0.0033	0.0097	0.2217	0.0597	0.1665	0.1059	0.1661	−0.1436	−0.0615	−0.1329
			0.2747	0.3186	0.2832	0.4701	0.2243	0.4516	0.2928	0.5305	0.2084	0.2708	0.2514
		b	0.0805	0.0064	0.0117	0.0972	0.0302	0.0642	0.0595	0.1133	−0.0421	0.0058	−0.0207
			0.0450	0.0543	0.0473	0.0841	0.0392	0.0643	0.0628	0.1333	0.0284	0.0689	0.0397
		β	0.2221	0.0066	0.0203	0.2508	0.0754	0.1604	0.1553	0.291	−0.1439	−0.0416	−0.1293
			0.3660	0.3892	0.3479	0.5846	0.2936	0.4357	0.4821	1.1013	0.2538	0.365	0.2984
		$\alpha_{1}$	1.2031	0.7733	0.6927	2.2615	0.6583	2.2476	1.1365	3.3897	−0.0646	0.6888	0.0467
			13.1938	20.6576	14.2873	89.274	8.2965	395.6931	29.4882	991.2837	3.0583	160.649	4.218
		$\alpha_{2}$	1.6083	1.1004	0.9721	3.2072	0.8946	3.1749	1.6272	5.7158	−0.0481	1.2016	0.1031
			24.9228	41.913	27.9622	203.2504	15.679	919.4307	70.086	3814.4497	5.2112	593.7171	7.2782

**Table 2:** The biases (first row) and the MSEs (second row) of the estimators for N=60.

*a*	*b*	Par	ML	LS	WLS	CM	AD	RTAD	LTAD	ADLTS	MPS	MSAD	MSALD
0.2	0.2	*a*	0.0479	−0.0294	−0.0124	0.0533	0.0001	0.0437	0.0108	−0.0029	−0.1092	−0.0583	−0.0952
			0.0612	0.0781	0.0669	0.0879	0.0617	0.1033	0.0666	0.1135	0.0644	0.0916	0.0871
		*b*	0.0065	−0.0013	0.0008	0.0086	0.0022	0.0067	0.0045	0.0074	−0.0088	0.0071	0.0009
			0.0006	0.0011	0.0009	0.0014	0.0008	0.0014	0.0010	0.0015	0.0005	0.0302	0.0109
		β	0.0982	−0.0204	0.0067	0.1037	0.0280	0.0733	0.0627	0.0910	−0.1440	−0.0556	−0.1151
			0.1539	0.1744	0.1564	0.2091	0.1478	0.1925	0.2051	0.5200	0.1377	0.2125	0.1737
		$\alpha_{1}$	0.1061	0.0155	0.0301	0.1354	0.0428	0.1346	0.0614	0.0788	−0.1018	−0.0181	−0.0694
			0.1404	0.1581	0.1364	0.2303	0.1254	0.2815	0.1458	0.2756	0.0927	0.1509	0.1235
		$\alpha_{2}$	0.1135	0.0177	0.0331	0.1459	0.0465	0.1449	0.0668	0.0876	−0.1073	−0.0183	−0.072
			0.1574	0.1783	0.1534	0.2620	0.1408	0.3195	0.1652	0.3176	0.1028	0.1614	0.1342
0.2	0.5	*a*	0.0612	−0.0198	−0.0029	0.0612	0.0133	0.0455	0.0307	0.0247	−0.0902	−0.0534	−0.0783
			0.0702	0.0914	0.0784	0.1035	0.0716	0.1148	0.0794	0.1190	0.0686	0.0787	0.083
		*b*	0.0199	0.001	0.0061	0.0261	0.0093	0.0184	0.0174	0.0258	−0.017	−0.008	−0.0149
			0.0052	0.0094	0.0078	0.0114	0.0070	0.0107	0.0083	0.0116	0.0042	0.0450	0.0151
		β	0.1126	−0.0017	0.0242	0.1207	0.0435	0.079	0.0893	0.1347	−0.1112	−0.0359	−0.0927
			0.1836	0.224	0.2012	0.2657	0.1851	0.2254	0.2498	0.5309	0.1534	0.2262	0.1954
		$\alpha_{1}$	0.1459	0.0471	0.0610	0.1816	0.0773	0.1742	0.1117	0.1433	−0.0785	−0.0188	−0.0514
			0.2178	0.2704	0.2299	0.3997	0.1972	0.7159	0.2449	0.4582	0.1305	0.1609	0.1669
		$\alpha_{2}$	0.1730	0.0611	0.0764	0.2205	0.0944	0.2111	0.1365	0.1804	−0.088	−0.0169	−0.0563
			0.2971	0.378	0.3194	0.5689	0.2708	1.0313	0.3421	0.6773	0.1727	0.2222	0.2222
0.2	0.8	*a*	0.0586	−0.0234	−0.0072	0.0563	0.0093	0.0405	0.0308	0.0061	−0.0986	−0.0454	−0.087
			0.0686	0.0812	0.0757	0.0913	0.0677	0.1053	0.0756	0.1081	0.0697	0.0838	0.0774
		*b*	0.0292	−0.0047	0.0030	0.0355	0.0103	0.0241	0.0258	0.0304	−0.0346	−0.0372	−0.0272
			0.0129	0.0203	0.0177	0.0243	0.0163	0.0256	0.0199	0.0253	0.0107	0.0987	0.0211
		β	0.0984	−0.0195	0.0028	0.1005	0.028	0.0626	0.0793	0.0801	−0.1347	−0.0618	−0.109
			0.1630	0.1808	0.1680	0.2118	0.1586	0.2018	0.2169	0.4106	0.1437	0.2012	0.1729
		$\alpha_{1}$	0.1557	0.0380	0.0602	0.1813	0.0774	0.1722	0.1219	0.1191	−0.0975	−0.0046	−0.0727
			0.2544	0.2838	0.2903	0.4122	0.2358	0.573	0.2792	0.4343	0.1556	0.2609	0.1739
		$\alpha_{2}$	0.2030	0.0569	0.0846	0.2436	0.1053	0.2328	0.1658	0.1724	−0.119	−0.0014	−0.0856
			0.4142	0.4749	0.4885	0.7077	0.3898	1.0139	0.4699	0.7788	0.2404	0.4407	0.2741
0.5	0.2	*a*	0.0784	−0.0112	0.0058	0.0818	0.0272	0.0634	0.0420	0.0217	−0.1033	−0.0491	−0.0836
			0.0885	0.1091	0.0951	0.1275	0.0881	0.1372	0.0940	0.1377	0.0838	0.1134	0.1031
		*b*	0.0089	0.0007	0.0026	0.0108	0.0044	0.008	0.0071	0.0087	−0.0074	0.0088	0.0032
			0.0008	0.0013	0.0011	0.0016	0.0010	0.0016	0.0011	0.0016	0.0007	0.0039	0.0031
		β	0.1144	0.0017	0.0238	0.1196	0.0478	0.083	0.0834	0.0921	−0.1128	−0.0422	−0.0831
			0.1543	0.1764	0.1551	0.2132	0.1465	0.1904	0.1868	0.3766	0.1297	0.1976	0.1664
		$\alpha_{1}$	0.2286	0.0894	0.1047	0.2846	0.1352	0.2600	0.1690	0.1806	−0.1081	0.0185	−0.0557
			0.4561	0.5343	0.4457	0.8315	0.4080	0.8892	0.4570	0.7311	0.2554	0.4291	0.3223
		$\alpha_{2}$	0.2443	0.0977	0.1135	0.3064	0.1455	0.2797	0.1822	0.1971	−0.1131	0.0214	−0.0570
			0.5157	0.6097	0.5062	0.9561	0.4620	1.0182	0.5189	0.8383	0.2855	0.4584	0.3503
0.5	0.8	*a*	0.0635	−0.0262	−0.0107	0.0646	0.0119	0.0558	0.0276	−0.0016	−0.1137	−0.0661	−0.1000
			0.0727	0.0900	0.0776	0.1035	0.0717	0.1251	0.0811	0.1121	0.0735	0.1014	0.0905
		*b*	0.0283	−0.0085	−0.0012	0.0319	0.0070	0.024	0.0185	0.0203	−0.0391	−0.0121	−0.0288
			0.0117	0.0181	0.0148	0.0218	0.0137	0.0222	0.0172	0.0208	0.0100	0.0293	0.0158
		β	0.0863	−0.0293	−0.0091	0.0846	0.0163	0.0601	0.0496	0.0372	−0.136	−0.0796	−0.1175
			0.1210	0.1445	0.1248	0.1699	0.1166	0.1616	0.1585	0.2766	0.1105	0.1556	0.1394
		$\alpha_{1}$	0.2227	0.0514	0.0695	0.2726	0.1096	0.2867	0.1594	0.1383	−0.1611	−0.0231	−0.1136
			0.4677	0.5160	0.4442	0.8094	0.4064	1.1540	0.5069	0.6768	0.2698	0.5499	0.3470
		$\alpha_{2}$	0.2879	0.0745	0.0957	0.3602	0.1450	0.3778	0.2134	0.1959	−0.1976	−0.0188	−0.1359
			0.7518	0.8406	0.7165	1.3573	0.6520	1.9303	0.8381	1.1296	0.4137	0.8814	0.5302
0.8	0.8	*a*	0.0829	−0.0135	0.0045	0.0908	0.0228	0.0666	0.0397	0.0342	−0.1135	−0.0656	−0.1007
			0.0911	0.1186	0.0989	0.1414	0.0896	0.1462	0.0985	0.1513	0.0861	0.1199	0.1089
		*b*	0.0349	−0.0003	0.0067	0.0413	0.0126	0.0282	0.0247	0.0361	−0.0372	−0.0016	−0.0269
			0.0144	0.0214	0.0178	0.0263	0.0161	0.0242	0.0198	0.0311	0.0116	0.0207	0.0153
		β	0.0979	−0.0056	0.013	0.1068	0.0310	0.0703	0.0645	0.0826	−0.1204	−0.0557	−0.1030
			0.1270	0.1553	0.1346	0.1874	0.1239	0.1673	0.1612	0.3091	0.1096	0.1616	0.1423
		$\alpha_{1}$	0.3912	0.1684	0.1810	0.5346	0.2137	0.4568	0.2853	0.3834	−0.1965	0.0052	−0.1242
			1.1806	1.7044	1.2545	2.9053	1.0389	2.5739	1.2745	2.4445	0.6066	1.1864	0.8375
		$\alpha_{2}$	0.5060	0.2354	0.246	0.7091	0.2835	0.6029	0.3817	0.533	−0.2375	0.0293	−0.1426
			1.9094	2.8927	2.0799	5.0970	1.6869	4.4135	2.1236	4.3668	0.9335	1.9385	1.2979

**Table 3:** The biases (first row) and the MSEs (second row) of the estimators for N=120.

*a*	*b*	Par	ML	LS	WLS	CM	AD	RTAD	LTAD	ADLTS	MPS	MSAD	MSALD
0.2	0.2	*a*	0.0285	−0.011	0.0014	0.0305	0.008	0.0244	0.0148	−0.0241	−0.0722	−0.0408	−0.0631
			0.0295	0.0385	0.0325	0.0408	0.0317	0.0479	0.0338	0.0547	0.0321	0.0463	0.0393
		*b*	0.0042	0.0006	0.002	0.0056	0.0026	0.0045	0.0038	0.002	−0.0045	0.0077	−0.0005
			0.0003	0.0006	0.0005	0.0007	0.0004	0.0007	0.0005	0.0006	0.0003	0.0081	0.0025
		β	0.0648	0.0059	0.0241	0.0682	0.033	0.0501	0.0536	0.0066	−0.0748	−0.0294	−0.0635
			0.0753	0.092	0.0794	0.1021	0.0762	0.094	0.101	0.2223	0.0698	0.1027	0.0852
		$\alpha_{1}$	0.0574	0.012	0.0242	0.0687	0.0322	0.0658	0.0427	0.0059	−0.0731	−0.0215	−0.0564
			0.0576	0.0713	0.0606	0.0855	0.0592	0.1027	0.065	0.0983	0.0464	0.0748	0.0573
		$\alpha_{2}$	0.0614	0.0137	0.0264	0.0742	0.0349	0.071	0.0463	0.008	−0.0767	−0.0207	−0.0586
			0.0643	0.0803	0.0681	0.0968	0.0664	0.116	0.0732	0.1109	0.0514	0.0806	0.0628
0.2	0.5	*a*	0.0246	−0.0118	−0.0008	0.0278	0.0014	0.0171	0.0093	−0.0245	−0.0725	−0.0413	−0.0597
			0.0280	0.0351	0.0299	0.0372	0.0286	0.0415	0.0327	0.0568	0.0319	0.0427	0.0381
		*b*	0.0095	0.0009	0.0041	0.0132	0.0045	0.0086	0.0078	0.0048	−0.0124	−0.0040	−0.0128
			0.0019	0.0035	0.0028	0.004	0.0027	0.004	0.0031	0.0038	0.0018	0.0155	0.0057
		β	0.0554	0.0023	0.0186	0.0627	0.0219	0.0373	0.0404	0.0023	−0.0794	−0.0445	−0.0692
			0.0704	0.0858	0.0755	0.0949	0.0721	0.0857	0.097	0.2076	0.0704	0.0965	0.0823
		$\alpha_{1}$	0.0567	0.0103	0.0223	0.07	0.0243	0.0572	0.0393	0.0106	−0.0796	−0.0272	−0.0579
			0.0628	0.0743	0.0636	0.0897	0.06	0.0972	0.0726	0.1276	0.0532	0.0817	0.0672
		$\alpha_{2}$	0.0673	0.0144	0.028	0.0846	0.0302	0.0692	0.0483	0.0174	−0.0901	−0.0289	−0.0657
			0.0830	0.1001	0.0852	0.1221	0.08	0.1316	0.0978	0.1738	0.0691	0.1075	0.0881
0.2	0.8	*a*	0.0311	−0.0063	0.0059	0.0329	0.0104	0.0226	0.0189	−0.0145	−0.0625	−0.0383	−0.0559
			0.0282	0.0386	0.0322	0.0411	0.0309	0.0476	0.0322	0.0508	0.0305	0.043	0.0371
		*b*	0.0133	−0.0014	0.0041	0.0182	0.0062	0.0112	0.0127	0.0083	−0.0237	−0.0148	−0.0211
			0.005	0.0094	0.0074	0.0103	0.007	0.0106	0.008	0.0105	0.0047	0.0234	0.0095
		β	0.0465	−0.0067	0.0101	0.0518	0.0178	0.0288	0.0405	0.0055	−0.0902	−0.0489	−0.0763
			0.0666	0.0843	0.072	0.0919	0.0691	0.0851	0.091	0.2065	0.0651	0.0960	0.0788
		$\alpha_{1}$	0.072	0.0231	0.0365	0.0886	0.0424	0.0773	0.058	0.0238	−0.0732	−0.0257	−0.058
			0.0769	0.1035	0.0861	0.1256	0.0813	0.1458	0.0874	0.1323	0.0628	0.0997	0.0766
		$\alpha_{2}$	0.0922	0.0322	0.0485	0.1167	0.0557	0.1017	0.0769	0.0384	−0.0911	−0.0299	−0.0714
			0.1197	0.1661	0.1368	0.2046	0.128	0.2364	0.1398	0.2168	0.0954	0.1571	0.1170
0.5	0.2	*a*	0.0342	−0.0083	0.0023	0.0380	0.0067	0.0267	0.0136	−0.0122	−0.0766	−0.0492	−0.0645
			0.0358	0.0498	0.0419	0.0538	0.0397	0.0597	0.0419	0.0614	0.0383	0.0537	0.0473
		*b*	0.0040	0.0002	0.0013	0.0052	0.0017	0.0035	0.0030	0.0025	−0.0059	0.0057	0.0005
			0.0003	0.0006	0.0005	0.0007	0.0005	0.0007	0.0005	0.0007	0.0003	0.0017	0.0009
		β	0.0529	−0.0004	0.0116	0.0579	0.0186	0.0362	0.0349	0.0135	−0.0868	−0.0488	−0.0743
			0.0656	0.0822	0.0709	0.0908	0.0682	0.0849	0.0819	0.1759	0.0696	0.0922	0.0798
		$\alpha_{1}$	0.0935	0.0321	0.0432	0.1195	0.0484	0.1045	0.0630	0.0360	−0.1014	−0.0372	−0.0714
			0.1320	0.1850	0.1523	0.2298	0.1399	0.2473	0.1508	0.2202	0.1029	0.1630	0.1313
		$\alpha_{2}$	0.0997	0.0352	0.0467	0.1283	0.0522	0.1121	0.0679	0.0405	−0.1067	−0.0369	−0.0739
			0.1478	0.2088	0.1714	0.2604	0.1571	0.2795	0.1696	0.2494	0.1146	0.1769	0.1426
0.5	0.8	*a*	0.0337	−0.0045	0.0082	0.0402	0.0118	0.0273	0.0219	−0.0216	−0.0658	−0.0385	−0.0643
			0.0347	0.0464	0.0400	0.0504	0.0377	0.0585	0.0411	0.0615	0.0377	0.0592	0.0447
		*b*	0.0181	0.0037	0.0095	0.0237	0.0102	0.0164	0.0167	0.0065	−0.0170	0.0051	−0.0153
			0.0060	0.0105	0.0088	0.0118	0.0080	0.0120	0.0094	0.0110	0.0059	0.0137	0.0079
		β	0.0562	0.0077	0.0245	0.0642	0.0275	0.0420	0.0473	−0.0036	−0.0608	−0.0246	−0.0610
			0.0647	0.0830	0.0732	0.0919	0.0681	0.0879	0.0874	0.1608	0.0662	0.0981	0.0754
		$\alpha_{1}$	0.1126	0.0477	0.0669	0.1498	0.0704	0.1310	0.0979	0.0302	−0.0944	−0.0083	−0.0843
			0.1861	0.2555	0.2202	0.3202	0.1948	0.3676	0.2276	0.3039	0.1469	0.2955	0.1726
		$\alpha_{2}$	0.1467	0.0680	0.0914	0.1991	0.0947	0.1743	0.1315	0.0506	−0.1129	0.0005	−0.1000
			0.2950	0.4221	0.3606	0.5361	0.3138	0.6072	0.3718	0.4952	0.2278	0.4655	0.2650
0.8	0.8	*a*	0.0494	−0.0073	0.0088	0.0426	0.0135	0.0266	0.0251	0.0124	−0.0632	−0.0462	−0.0631
			0.0425	0.0565	0.0463	0.0615	0.0431	0.0666	0.0487	0.0759	0.0406	0.0578	0.0499
		*b*	0.0194	−0.0003	0.006	0.0196	0.0072	0.0122	0.0139	0.017	−0.0193	−0.0033	−0.0190
			0.0068	0.0105	0.0086	0.0116	0.0081	0.0116	0.0095	0.0136	0.0064	0.0082	0.0080
		β	0.0553	−0.0033	0.0137	0.0506	0.0179	0.0302	0.0366	0.0335	−0.0622	−0.0410	−0.0661
			0.0615	0.077	0.0656	0.0841	0.0625	0.0806	0.0773	0.1491	0.0593	0.0766	0.0690
		$\alpha_{1}$	0.2076	0.0712	0.1001	0.2255	0.1072	0.1897	0.1517	0.1637	−0.1174	−0.0398	−0.1046
			0.4455	0.5891	0.4796	0.7507	0.4272	0.908	0.512	0.8034	0.2946	0.4755	0.3641
		$\alpha_{2}$	0.2663	0.0992	0.1337	0.2957	0.1416	0.2504	0.2006	0.2255	−0.1414	−0.0393	−0.1253
			0.7112	0.9563	0.7743	1.2341	0.6854	1.5122	0.8334	1.3339	0.4583	0.7325	0.5617

**Table 4:** The estimated shape parameter under $S_{u}=0.1$.

*a*	*b*	ML	LS	WLS	CM	AD	RTAD	LTAD	ADLTS	MPS	MSAD	MSALD
N=30												
0.2	0.2	1.5764	1.3741	1.4069	1.8315	1.4318	1.6450	1.5155	1.6306	1.1784	1.2928	1.2102
0.2	0.5	1.5989	1.4223	1.4054	1.7673	1.4316	1.7737	1.5195	1.6982	1.1973	1.3816	1.2422
0.2	0.8	1.6467	1.551	1.5229	2.0228	1.4835	1.7550	1.6445	2.0089	1.2271	1.3685	1.2551
0.5	0.2	2.1481	1.9522	1.9193	2.5690	1.9435	2.6801	2.053	2.2261	1.5502	1.7290	1.6069
0.5	0.8	2.3280	2.059	1.9889	2.6961	2.0586	2.5963	2.2125	2.5289	1.6719	1.8476	1.7170
0.8	0.8	3.4507	3.0582	2.9949	4.3113	2.9751	4.3038	3.361	5.0398	2.3420	2.9364	2.4377
N=60												
0.2	0.2	1.3487	1.2605	1.2748	1.3765	1.2871	1.3758	1.3049	1.3207	1.1469	1.2286	1.1780
0.2	0.5	1.4180	1.3252	1.3384	1.4486	1.3539	1.4422	1.385	1.4114	1.2100	1.2652	1.2352
0.2	0.8	1.4592	1.3539	1.3737	1.4791	1.3893	1.4710	1.4273	1.4212	1.2351	1.3191	1.2565
0.5	0.2	1.9031	1.7675	1.7825	1.9563	1.8124	1.9327	1.8448	1.8548	1.5764	1.6992	1.6270
0.5	0.8	1.9816	1.8285	1.8451	2.0228	1.8812	2.0354	1.9235	1.9013	1.6409	1.7623	1.6827
0.8	0.8	2.7543	2.5528	2.5658	2.8744	2.5961	2.8080	2.6569	2.7346	2.2326	2.4076	2.2956
N=120												
0.2	0.2	1.3015	1.2574	1.2692	1.3122	1.2770	1.3094	1.2871	1.2510	1.1748	1.2243	1.1908
0.2	0.5	1.3361	1.2926	1.3038	1.3476	1.3057	1.336	1.3194	1.2917	1.2093	1.2579	1.2298
0.2	0.8	1.3867	1.3426	1.3548	1.4002	1.3600	1.3904	1.3734	1.3412	1.2576	1.2998	1.2711
0.5	0.2	1.7728	1.7039	1.7199	1.7871	1.7300	1.7734	1.746	1.7195	1.5856	1.6495	1.6116
0.5	0.8	1.9058	1.8367	1.8523	1.9261	1.8575	1.9199	1.8781	1.8479	1.6924	1.7744	1.7346
0.8	0.8	2.5939	2.4710	2.4973	2.6074	2.5040	2.5757	2.5425	2.5496	2.3041	2.3716	2.3156

**Table 5:** The estimated reliability function $S_{u}=0.1$ for N=30.

*a*	*b*	*x*_0_	ML	LS	WLS	CM	AD	RTAD	LTAD	ADLTS	MPS	MSAD	MSALD
0.2	0.2	0.25	0.8369	0.8153	0.8175	0.838	0.8247	0.8341	0.8263	0.8187	0.7999	0.8056	0.7979
		0.5	0.6214	0.6113	0.6122	0.6222	0.6149	0.6242	0.6115	0.5964	0.6031	0.6011	0.6003
		1.25	0.1784	0.202	0.1988	0.1815	0.192	0.1888	0.1881	0.1862	0.2133	0.2087	0.2104
		2.50	0.0198	0.0302	0.0284	0.0215	0.0247	0.024	0.0241	0.0281	0.0347	0.0324	0.0365
0.2	0.5	0.25	0.8427	0.8177	0.8205	0.8395	0.8279	0.8341	0.8306	0.8253	0.8051	0.8142	0.8069
		0.5	0.6296	0.6173	0.6186	0.628	0.6229	0.6313	0.6177	0.6061	0.6099	0.6165	0.6119
		1.25	0.1856	0.2069	0.2044	0.1866	0.1982	0.1946	0.1919	0.1878	0.2198	0.2162	0.2199
		2.50	0.0199	0.0304	0.0285	0.0221	0.0248	0.0239	0.0246	0.0286	0.0342	0.0335	0.0358
0.2	0.8	0.25	0.8502	0.8266	0.8288	0.8485	0.8363	0.8437	0.8391	0.8337	0.8132	0.8237	0.8139
		0.5	0.6395	0.6275	0.6281	0.6398	0.6316	0.6443	0.628	0.6143	0.6199	0.6269	0.621
		1.25	0.1922	0.2134	0.2113	0.1931	0.2048	0.2041	0.1966	0.1894	0.2246	0.2213	0.2266
		2.50	0.0204	0.031	0.029	0.0228	0.0251	0.0245	0.0247	0.0278	0.0354	0.0336	0.0367
0.5	0.2	0.25	0.9113	0.8887	0.8911	0.9091	0.8991	0.9039	0.9017	0.8983	0.8761	0.8825	0.8756
		0.5	0.7357	0.7168	0.7185	0.7353	0.7243	0.733	0.7237	0.7141	0.7063	0.7086	0.7057
		1.25	0.2303	0.2528	0.2497	0.2335	0.2433	0.2427	0.2347	0.2233	0.2641	0.2591	0.2645
		2.50	0.0247	0.0361	0.0342	0.0267	0.0303	0.0294	0.0294	0.0329	0.0414	0.0385	0.0425
0.5	0.8	0.25	0.9215	0.8997	0.9021	0.9193	0.9095	0.9134	0.9123	0.9096	0.8879	0.8935	0.8865
		0.5	0.7550	0.7362	0.7383	0.7562	0.7428	0.7532	0.7421	0.7314	0.7225	0.7295	0.7244
		1.25	0.2482	0.2672	0.2655	0.2483	0.2607	0.2597	0.2526	0.2463	0.2807	0.2741	0.2764
		2.50	0.0258	0.0367	0.0351	0.0273	0.0316	0.0308	0.0302	0.0342	0.0422	0.0409	0.0433
0.8	0.8	0.25	0.9659	0.9487	0.9511	0.9625	0.9574	0.958	0.9591	0.9575	0.9415	0.9448	0.9398
		0.5	0.8522	0.8269	0.8307	0.8485	0.8381	0.8446	0.8399	0.8355	0.8138	0.8224	0.818
		1.25	0.3185	0.3339	0.3329	0.3175	0.3289	0.3298	0.3175	0.3029	0.3462	0.3352	0.3429
		2.50	0.0338	0.0477	0.0456	0.0368	0.041	0.0392	0.0398	0.0414	0.0532	0.0507	0.0541

**Table 6:** The estimated reliability function $S_{u}=0.1$ for N=60.

*a*	*b*	*x*_0_	ML	LS	WLS	CM	AD	RTAD	LTAD	ADLTS	MPS	MSAD	MSALD
0.2	0.2	0.25	0.8283	0.8166	0.8193	0.8284	0.8209	0.8273	0.8212	0.8166	0.8061	0.8112	0.8067
		0.5	0.6214	0.6155	0.6167	0.6211	0.6185	0.624	0.6151	0.6078	0.6114	0.6116	0.61
		1.25	0.1833	0.1946	0.1916	0.1838	0.1896	0.1885	0.1851	0.186	0.204	0.2003	0.2056
		2.50	0.0173	0.0222	0.0207	0.0184	0.0197	0.0191	0.0197	0.0243	0.0249	0.0244	0.0256
0.2	0.5	0.25	0.8398	0.8265	0.8293	0.838	0.8323	0.8359	0.8335	0.8292	0.8173	0.8203	0.8184
		0.5	0.6267	0.6216	0.6225	0.6273	0.6229	0.6296	0.621	0.6136	0.615	0.6165	0.6144
		1.25	0.1868	0.1993	0.1961	0.1886	0.1942	0.1931	0.1911	0.1928	0.2079	0.2068	0.2087
		2.50	0.0184	0.0233	0.022	0.0194	0.0209	0.0202	0.0212	0.0273	0.0265	0.0256	0.0266
0.2	0.8	0.25	0.8487	0.8355	0.8383	0.847	0.841	0.8447	0.8428	0.8373	0.8252	0.8323	0.826
		0.5	0.648	0.6415	0.6429	0.648	0.6435	0.6488	0.6427	0.6338	0.6324	0.6392	0.6338
		1.25	0.194	0.2054	0.2028	0.1947	0.2007	0.1998	0.1967	0.1979	0.2152	0.2112	0.2144
		2.50	0.0186	0.0233	0.022	0.0195	0.021	0.0207	0.0205	0.0254	0.0265	0.0259	0.0276
0.5	0.2	0.25	0.9089	0.8965	0.8992	0.9072	0.9024	0.9046	0.9032	0.8985	0.8862	0.8912	0.8874
		0.5	0.7299	0.72	0.7222	0.7299	0.7238	0.7304	0.7229	0.7161	0.7087	0.713	0.7114
		1.25	0.2366	0.2482	0.2457	0.2383	0.2436	0.2422	0.2398	0.2363	0.2555	0.2524	0.2559
		2.50	0.0235	0.0289	0.0275	0.0245	0.0264	0.0257	0.0264	0.0314	0.033	0.0323	0.0328
0.5	0.8	0.25	0.9205	0.9089	0.9113	0.9192	0.9145	0.917	0.9156	0.9106	0.8989	0.9033	0.8995
		0.5	0.7524	0.7401	0.743	0.7499	0.7457	0.7505	0.7448	0.7392	0.7321	0.7376	0.7349
		1.25	0.2493	0.2598	0.2575	0.2499	0.2555	0.255	0.2507	0.2488	0.2691	0.2647	0.2689
		2.50	0.0247	0.0301	0.0287	0.0255	0.0277	0.0274	0.0268	0.0313	0.0335	0.033	0.035
0.8	0.8	0.25	0.9664	0.9575	0.9599	0.9648	0.9618	0.9622	0.9626	0.9603	0.9515	0.9529	0.951
		0.5	0.8488	0.8352	0.8385	0.8474	0.8408	0.845	0.8428	0.8369	0.8247	0.8301	0.8267
		1.25	0.3231	0.332	0.33	0.3243	0.3293	0.3291	0.3244	0.3208	0.3382	0.338	0.3416
		2.50	0.0324	0.0386	0.037	0.0335	0.0356	0.0352	0.0351	0.0394	0.0434	0.0421	0.0439

**Table 7:** The estimated reliability function $S_{u}=0.1$ for N=120.

*a*	*b*	$x_{0}$	ML	LS	WLS	CM	AD	RTAD	LTAD	ADLTS	MPS	MSAD	MSALD
0.2	0.2	0.25	0.8275	0.8214	0.8234	0.8275	0.8244	0.8269	0.8246	0.8189	0.8113	0.8156	0.8125
		0.5	0.6188	0.6154	0.6164	0.6183	0.6173	0.6197	0.6149	0.6116	0.6136	0.6137	0.6129
		1.25	0.1833	0.1891	0.187	0.1835	0.1864	0.1862	0.1858	0.1922	0.1942	0.1931	0.1955
		2.50	0.0176	0.0199	0.0191	0.018	0.0188	0.0186	0.0186	0.0243	0.022	0.0223	0.0221
0.2	0.5	0.25	0.8364	0.8306	0.8325	0.8365	0.8328	0.8353	0.8335	0.8281	0.8207	0.8259	0.823
		0.5	0.629	0.6263	0.6271	0.6291	0.6269	0.631	0.6254	0.622	0.6218	0.6229	0.6202
		1.25	0.1874	0.1935	0.1912	0.188	0.1909	0.1906	0.1892	0.1961	0.2001	0.1998	0.2011
		2.50	0.0184	0.0212	0.0201	0.0193	0.0199	0.0196	0.02	0.024	0.0225	0.0227	0.023
0.2	0.8	0.25	0.8476	0.8413	0.8435	0.8472	0.8441	0.8457	0.8447	0.8397	0.8332	0.8356	0.8333
		0.5	0.6406	0.6381	0.6386	0.6412	0.6386	0.6422	0.6383	0.6342	0.634	0.6332	0.6325
		1.25	0.1938	0.2	0.1978	0.1947	0.1975	0.1969	0.1956	0.2001	0.2052	0.2047	0.2062
		2.50	0.0182	0.0207	0.0198	0.0188	0.0196	0.0193	0.0192	0.0238	0.0224	0.0229	0.0233
0.5	0.2	0.25	0.9083	0.9013	0.9036	0.9070	0.9047	0.9057	0.9053	0.9012	0.894	0.8968	0.8948
		0.5	0.728	0.7232	0.7244	0.7283	0.7253	0.7283	0.7254	0.7206	0.7159	0.7181	0.7185
		1.25	0.2404	0.2466	0.2443	0.2414	0.2441	0.2436	0.2413	0.2457	0.2537	0.2505	0.2513
		2.50	0.0228	0.0257	0.0245	0.0235	0.0244	0.024	0.0241	0.0295	0.028	0.0276	0.0282
0.5	0.8	0.25	0.921	0.9148	0.9167	0.9200	0.9174	0.9188	0.9181	0.9148	0.9069	0.9103	0.9091
		0.5	0.7494	0.7439	0.7453	0.7493	0.7459	0.7483	0.746	0.7413	0.738	0.7394	0.7388
		1.25	0.252	0.2578	0.256	0.2528	0.2557	0.2551	0.2539	0.2574	0.2634	0.2609	0.2628
		2.50	0.0248	0.0276	0.0266	0.0254	0.0263	0.0261	0.026	0.0304	0.0303	0.0298	0.0303
0.8	0.8	0.25	0.9671	0.9619	0.9637	0.9655	0.9643	0.9639	0.9648	0.9629	0.9583	0.9583	0.9578
		0.5	0.8467	0.8398	0.8425	0.8462	0.8427	0.8449	0.8432	0.8394	0.8312	0.8329	0.8328
		1.25	0.3248	0.3283	0.3271	0.3242	0.3276	0.3278	0.325	0.3287	0.3339	0.3325	0.3335
		2.50	0.0328	0.0363	0.0348	0.0336	0.0348	0.0344	0.0341	0.0383	0.0391	0.0380	0.0394

**Table 8:** Failure times of the capacitors.

Stress	Failure Data									
145 °C	0.075	0.359	0.701	0.722	0.738	1.015	1.388	2.285	3.157	3.547
	3.986	4.077	5.447	5.735	5.869	6.242	7.804	8.031	8.292	8.506
	8.584	11.512	12.370	16.062	17.790	19.767	20.145	21.971	30.438	42.004
240 °C	0.179	0.407	0.466	0.571	0.755	0.768	1.006	1.094	1.104	1.493
	1.494	2.877	3.001	3.160	3.283	4.654	5.259	5.925	6.229	6.462
	6.629	6.855	6.983	7.387	7.564	7.783	10.067	11.846	13.285	28.762
305 °C	0.116	0.189	0.300	0.305	0.314	0.403	0.433	0.440	0.468	0.609
	0.634	0.640	0.644	0.699	0.781	0.813	0.860	1.009	1.176	1.184
	1.245	2.071	2.189	2.288	2.637	2.841	2.910	2.954	3.111	4.617

**Table 9:** Goodness-of-fit results for different distributions for each stress levels.

Stress	Model	${\hat{a}}_{ML}$	${\hat{\beta }}_{ML}$	KS	*p*-Value	RMSE	*R* ^2^	AIC	BIC
145 °C	EHL	0.7024	0.1186	0.0898	0.9610	0.0324	0.9876	197.4718	200.2742
	GL	3.2931	5.4430	0.1386	0.5791	0.0724	0.9457	214.7454	217.5478
	ER	0.3168	0.0023	0.1202	0.7521	0.0411	0.9788	198.2941	201.0965
	GIL	0.5611	2.1096	0.1580	0.4105	0.0883	0.8785	215.5364	218.3388
	EL	1.2665	0.2171	0.1869	0.2201	0.1153	0.9000	213.0956	215.8980
240 °C	EHL	0.8066	0.2301	0.1151	0.7971	0.0499	0.9717	164.1819	166.9843
	GL	3.4320	3.0070	0.1591	0.4012	0.0663	0.9534	178.2044	181.0068
	ER	0.3398	0.0073	0.1346	0.6171	0.0582	0.9548	167.3063	170.1087
	GIL	0.7546	1.9412	0.1540	0.4429	0.0776	0.9255	172.2434	175.0458
	EL	1.7689	0.4126	0.2836	0.0125	0.1302	0.8790	183.4953	186.2977
305 °C	EHL	1.1092	1.1219	0.1378	0.5868	0.0745	0.9428	79.0451	81.8475
	GL	3.9172	0.6818	0.1719	0.3086	0.0983	0.9117	88.4685	91.2709
	ER	0.4725	0.1980	0.1585	0.4062	0.0818	0.9279	79.9938	82.7962
	GIL	0.9841	0.9069	0.0824	0.9828	0.0398	0.9792	80.8900	83.6924
	EL	5.4143	1.9481	0.3961	0.0001	0.2360	0.7288	124.7059	127.5083

**Table 10:** The different estimates (first row) and ESE (second row) for the real dataset.

Parameter	ML	LS	WLS	CM	AD	RTAD	LTAD	ADLTS	MPS	MSAD	MSALD
*a*	0.7171	1.4036	2.4437	1.6116	0.9596	1.3563	0.9154	0.5460	0.4914	0.5775	0.5283
	0.4104	0.5922	0.8474	0.6339	0.4462	0.5641	0.4675	0.4458	0.2998	0.3813	0.3382
*b*	−0.0051	−0.0082	−0.0108	−0.0087	−0.0069	−0.0087	−0.0061	−0.0042	−0.0046	−0.0047	−0.0046
	0.0014	0.0019	0.0028	0.0021	0.0015	0.0018	0.0016	0.0016	0.0010	0.0012	0.0012
β	0.1885	0.2014	0.3158	0.2210	0.1655	0.1650	0.2009	0.2336	0.1613	0.1677	0.1490
	0.0354	0.0438	0.0631	0.0471	0.0345	0.0372	0.0478	0.2163	0.0249	0.0407	0.0291
$\alpha_{1}$	0.9821	1.2389	2.4091	1.4113	0.9584	1.1005	1.0242	0.9402	0.8346	0.9002	0.8659
	0.2456	0.4515	2.3838	0.5536	0.2526	0.3911	0.2902	0.3919	0.1423	0.2164	0.1816
$\alpha_{2}$	0.6067	0.5684	0.8644	0.6153	0.4970	0.4818	0.5711	0.6315	0.5373	0.5755	0.5574
	0.0747	0.1007	0.2389	0.1147	0.0694	0.0855	0.0820	0.2383	0.0486	0.0919	0.0849
$\alpha_{3}$	0.4364	0.3335	0.4287	0.3486	0.3171	0.2738	0.3829	0.4809	0.3975	0.4238	0.4124
	0.0504	0.0424	0.0580	0.0454	0.0399	0.0369	0.0526	0.1981	0.0330	0.0630	0.0688

**Table 11:** Different estimates of MTTF (first row) and the corresponding ESEs (second row) for the real dataset.

Stress	ML	LS	WLS	CM	AD	RTAD	LTAD	ADLTS	MPS	MSAD	MSALD
145 °C	7.2764	7.7840	6.8898	7.6099	8.1685	8.8873	6.9970	5.7202	7.6955	7.7593	8.5231
	0.0023	0.0057	0.0002	0.0052	0.0087	0.0084	0.0047	0.0024	0.0055	0.0136	0.0099
240 °C	5.3383	4.7757	4.0183	4.5975	5.2815	5.1793	4.8013	4.4276	5.7289	5.7851	6.3644
	0.0002	0.0041	0.0008	0.0038	0.0056	0.0065	0.0022	0.0010	0.0040	0.0099	0.0123
305 °C	4.2125	3.2060	2.4812	3.0253	3.7487	3.3387	3.5786	3.6527	4.5871	4.6325	5.1056
	0.0007	0.0035	0.0014	0.0034	0.0044	0.0057	0.0013	0.0023	0.0035	0.0081	0.0136

**Table 12:** Different estimates of $R\left(x\right)$, $\alpha_{u}$ and the corresponding ESEs under usual stress $S_{u}=125$.

$x_{0}$	ML	LS	WLS	CM	AD	RTAD	LTAD	ADLTS	MPS	MSAD	MSALD
828	0.9376	0.9735	0.9978	0.9821	0.9478	0.9703	0.9440	0.9085	0.9162	0.9286	0.9292
	0.0001	0.0004	0.0002	0.0004	0.0003	0.0004	0.0003	0.0003	0.0003	0.0001	0.0002
2042	0.8354	0.9027	0.9693	0.9204	0.8602	0.9041	0.8429	0.7735	0.8098	0.8271	0.8341
	$4\times 10^{-5}$	0.0005	0.0005	0.0006	0.0003	0.0005	0.0003	0.0008	0.0002	$3\times 10^{-5}$	0.0001
4620	0.6207	0.7041	0.7573	0.7186	0.6705	0.7341	0.6202	0.5151	0.6113	0.6269	0.6499
	0.0001	0.0002	0.0002	0.0002	0.0001	0.0002	0.0001	0.0008	$4\times 10^{-5}$	0.0003	0.0001
6016	0.5158	0.5921	0.5939	0.5980	0.5742	0.6392	0.5099	0.4008	0.5182	0.5305	0.5609
	$2\times 10^{-5}$	0.0001	$4\times 10^{-5}$	0.0001	0.0001	0.0002	0.0001	0.0006	$10\times 10^{-6}$	0.0004	0.0001
${\hat{\alpha }}^{*}$	1.0869	1.4598	2.9893	1.6808	1.1004	1.3094	1.1582	1.0224	0.9157	0.9891	0.9501
	0.0022	0.0019	0.0260	0.0025	$4\times 10^{-5}$	0.0001	0.0013	0.0046	0.0007	0.0010	$2\times 10^{-5}$

**Table 13:** Lengths of the 95% percentile bootstrap confidence intervals for different estimation methods.

Par	ML	LS	WLS	CM	AD	RTAD	LTAD	ADLTS	MPS	MSAD	MSALD
a	1.6090	2.3508	3.2223	2.4857	1.7892	2.2230	1.8165	1.8059	1.1197	1.3712	1.2942
b	0.0056	0.0076	0.0106	0.0081	0.0060	0.0072	0.0063	0.0066	0.0040	0.0047	0.0048
β	0.1411	0.1677	0.2498	0.1805	0.1338	0.1433	0.1762	0.8636	0.0929	0.1357	0.0977
$\alpha_{1}$	0.9384	1.7767	5.6674	2.1377	0.9949	1.5289	1.1268	1.4693	0.5235	0.7355	0.6715
$\alpha_{2}$	0.2873	0.3752	0.8175	0.4337	0.2657	0.3225	0.3335	0.9292	0.1839	0.3428	0.3338
$\alpha_{3}$	0.1933	0.1681	0.2319	0.1815	0.1544	0.1486	0.2031	0.7890	0.1263	0.2337	0.2531
$\alpha_{u}$	1.1697	2.3761	8.2300	2.8559	1.2843	2.0320	1.4192	1.7564	0.6423	0.8751	0.7996
$R_{1}$	0.1353	0.1401	0.0562	0.1277	0.1461	0.1321	0.1578	0.2126	0.1165	0.1268	0.1173
$R_{2}$	0.2481	0.2584	0.1988	0.2507	0.2427	0.2388	0.2807	0.5037	0.1813	0.2164	0.1907
$R_{3}$	0.3402	0.3887	0.5165	0.4067	0.3255	0.3525	0.4010	0.7170	0.2531	0.2913	0.2656
$R_{4}$	0.3488	0.4194	0.6053	0.4452	0.3626	0.3842	0.4217	0.6261	0.2507	0.3199	0.2777
$MTTF_{1}$	4.7971	5.8878	5.1684	5.7018	5.9633	6.5214	5.7015	8.0586	4.0444	5.5857	4.9488
$MTTF_{2}$	2.7244	2.7568	2.0750	2.5511	3.1133	3.0864	3.0658	5.3149	2.4231	3.0915	3.1113
$MTTF_{3}$	2.1341	2.0633	1.2214	1.9352	2.1902	2.2002	2.1588	4.0424	1.9470	2.9141	2.8451

## Data Availability

All data generated or analyzed during this study are included in this published article.

## Supplementary Materials

The following supporting information can be downloaded at https://www.mdpi.com/article/10.3390/e28101140/s1, Table S1: The biases and the MSEs of parameter estimates under stress-dependent scale parameters N=30; Table S2: The biases and the MSEs of parameter estimates under stress-dependent scale parameters N=60; Table S3: The biases and the MSEs of parameter estimates under stress-dependent scale parameters N=120; Table S4: The biases and the MSEs of estimators for $n_{1}=20$, $n_{2}=40$ under two accelerated stress levels; Table S5: The biases and the MSEs of estimators for $n_{1}=10$, $n_{2}=20$ under two accelerated stress levels; Table S6: The biases and the MSEs of estimators under three accelerated stress levels; Table S7: Summary of the best estimation methods based on bias and MSE; Table S8: Summary of the estimation methods considered in the study.

## Abbreviations

The following abbreviations are used in this manuscript:

AD	Anderson Darling
ADLTS	Anderson Darling Left-Tail Second Order
AIC	Akaike Information Criterion
ALT	Accelerated Life Testing
cdf	Cumulative Distribution Function
CM	Cramér-von Mises
CSALT	Constant Stress
EHL	Exponentiated Half Logistic
ESE	Estimated Standard Errors
hrf	Hazard Rate Function
KS	Kolmogorov–Smirnov
LS	Least Squares
LTAD	Left-Tail Anderson Darling
ML	Maximum Likelihood
MPS	Maximum Product of Spacing
MRE	Mean Relative Estimates
MSAD	Minimum Spacing Absolute Distance
MSALD	Minimum Spacing Absolute-Log Distance
MSE	Mean Square Error
MTTF	Mean Time To Failure
pdf	Probability Density Function
PWM	Probability-Weighted Moments
RMSE	Root Mean Squared Error
RTAD	Right-Tail Anderson Darling
WLS	Weighted Least Squares

## Appendix A. The ML Equations

$$\frac{\partial l\left(a,b,\beta \right)}{\partial a}=\sum_{j=1}^{k}\sum_{i=1}^{n_{j}}\left[1+e^{a+bS_{j}}ln\left(\frac{1-e^{-\beta x_{ij}}}{1+e^{-\beta x_{ij}}}\right)\right]=0,$$

$$\frac{\partial l\left(a,b,\beta \right)}{\partial b}=\sum_{j=1}^{k}\sum_{i=1}^{n_{j}}\left[S_{j}+S_{j}e^{a+bS_{j}}ln\left(\frac{1-e^{-\beta x_{ij}}}{1+e^{-\beta x_{ij}}}\right)\right]=0,$$

$$\frac{\partial l\left(a,b,\beta \right)}{\partial \beta }=\frac{N}{\beta }+\sum_{j=1}^{k}\sum_{i=1}^{n_{j}}\left[\left(e^{a+bS_{j}}-1\right)\frac{2x_{ij}e^{-\beta x_{ij}}}{1-e^{-2\beta x_{ij}}}-x_{ij}+2\frac{x_{ij}e^{-\beta x_{ij}}}{1+e^{-\beta x_{ij}}}\right]=0.$$

## Appendix B. The LS Equations

$$\frac{\partial S\left(a,b,\beta \right)}{\partial a}=\sum_{j=1}^{k}\sum_{i=1}^{n_{j}}\left[{\left(\frac{1-e^{-\beta x_{ij}}}{1+e^{-\beta x_{ij}}}\right)}^{e^{a+bS_{j}}}-\frac{i}{n_{j}+1}\right]\Delta_{1ij}=0,$$

$$\frac{\partial S\left(a,b,\beta \right)}{\partial b}=\sum_{j=1}^{k}\sum_{i=1}^{n_{j}}\left[{\left(\frac{1-e^{-\beta x_{ij}}}{1+e^{-\beta x_{ij}}}\right)}^{e^{a+bS_{j}}}-\frac{i}{n_{j}+1}\right]\Delta_{2ij}=0,$$

$$\frac{\partial S\left(a,b,\beta \right)}{\partial \beta }=\sum_{j=1}^{k}\sum_{i=1}^{n_{j}}\left[{\left(\frac{1-e^{-\beta x_{ij}}}{1+e^{-\beta x_{ij}}}\right)}^{e^{a+bS_{j}}}-\frac{i}{n_{j}+1}\right]\Delta_{3ij}=0,$$

where

$$\Delta_{1ij}=e^{a+bS_{j}}ln\left(\frac{1-e^{-\beta x_{ij}}}{1+e^{-\beta x_{ij}}}\right){\left(\frac{1-e^{-\beta x_{ij}}}{1+e^{-\beta x_{ij}}}\right)}^{e^{a+bS_{j}}},$$

$$\Delta_{2ij}=S_{j}e^{a+bS_{j}}ln\left(\frac{1-e^{-\beta x_{ij}}}{1+e^{-\beta x_{ij}}}\right){\left(\frac{1-e^{-\beta x_{ij}}}{1+e^{-\beta x_{ij}}}\right)}^{e^{a+bS_{j}}},$$

$$\Delta_{3ij}=e^{a+bS_{j}}{\left(\frac{1-e^{-\beta x_{ij}}}{1+e^{-\beta x_{ij}}}\right)}^{e^{a+bS_{j}}-1}\left(\frac{2x_{ij}e^{-\beta x_{ij}}}{{\left(1+e^{-\beta x_{ij}}\right)}^{2}}\right).$$

## Appendix C. The WLS Equations

$$\frac{\partial W\left(a,b,\beta \right)}{\partial a}=\sum_{j=1}^{k}\sum_{i=1}^{n_{j}}\frac{{\left(n_{j}+1\right)}^{2}\left(n_{j}+2\right)}{i\left(n_{j}-i+1\right)}\left[{\left(\frac{1-e^{-\beta x_{ij}}}{1+e^{-\beta x_{ij}}}\right)}^{e^{a+bS_{j}}}-\frac{i}{n_{j}+1}\right]\Delta_{1ij}=0,$$

$$\frac{\partial W\left(a,b,\beta \right)}{\partial b}=\sum_{j=1}^{k}\sum_{i=1}^{n_{j}}\frac{{\left(n_{j}+1\right)}^{2}\left(n_{j}+2\right)}{i\left(n_{j}-i+1\right)}\left[{\left(\frac{1-e^{-\beta x_{ij}}}{1+e^{-\beta x_{ij}}}\right)}^{e^{a+bS_{j}}}-\frac{i}{n_{j}+1}\right]\Delta_{2ij}=0,$$

$$\frac{\partial W\left(a,b,\beta \right)}{\partial \beta }=\sum_{j=1}^{k}\sum_{i=1}^{n_{j}}\frac{{\left(n_{j}+1\right)}^{2}\left(n_{j}+2\right)}{i\left(n_{j}-i+1\right)}\left[{\left(\frac{1-e^{-\beta x_{ij}}}{1+e^{-\beta x_{ij}}}\right)}^{e^{a+bS_{j}}}-\frac{i}{n_{j}+1}\right]\Delta_{3ij}=0,$$

where $\Delta_{1ij},\Delta_{2ij}$ and $\Delta_{3ij}$ are specified in Appendix B.

## Appendix D. The CM Equations

$$\frac{\partial C\left(a,b,\beta \right)}{\partial a}=\sum_{j=1}^{k}\sum_{i=1}^{n_{j}}\left[{\left(\frac{1-e^{-\beta x_{ij}}}{1+e^{-\beta x_{ij}}}\right)}^{e^{a+bS_{j}}}-\frac{2i-1}{2n_{j}}\right]\Delta_{1ij}=0,$$

$$\frac{\partial C\left(a,b,\beta \right)}{\partial b}=\sum_{j=1}^{k}\sum_{i=1}^{n_{j}}\left[{\left(\frac{1-e^{-\beta x_{ij}}}{1+e^{-\beta x_{ij}}}\right)}^{e^{a+bS_{j}}}-\frac{2i-1}{{2n}_{j}}\right]\Delta_{2ij}=0,$$

$$\frac{\partial C\left(a,b,\beta \right)}{\partial \beta }=\sum_{j=1}^{k}\sum_{i=1}^{n_{j}}\left[{\left(\frac{1-e^{-\beta x_{ij}}}{1+e^{-\beta x_{ij}}}\right)}^{e^{a+bS_{j}}}-\frac{2i-1}{{2n}_{j}}\right]\Delta_{3ij}=0,$$

where $\Delta_{1ij},\Delta_{2ij}$ and $\Delta_{3ij}$ are specified in Appendix B.

## Appendix E. The AD Equations

$$\frac{\partial A\left(a,b,\beta \right)}{\partial a}=\sum_{j=1}^{k}\sum_{i=1}^{n_{j}}\frac{2i-1}{n_{j}}\left[\frac{\Delta_{1ij}}{F\left(x_{ij};a,b,\beta \right)}-\frac{\Delta_{{1n}_{j}+1-ij}}{1-F\left(x_{n_{j}+1-ij};a,b,\beta \right)}\right]=0,$$

$$\frac{\partial A\left(a,b,\beta \right)}{\partial b}=\sum_{j=1}^{k}\sum_{i=1}^{n_{j}}\frac{2i-1}{n_{j}}\left[\frac{\Delta_{2ij}}{F\left(x_{ij};a,b,\beta \right)}-\frac{\Delta_{{2n}_{j}+1-ij}}{1-F\left(x_{n_{j}+1-ij};a,b,\beta \right)}\right]=0,$$

$$\frac{\partial A\left(a,b,\beta \right)}{\partial \beta }=\sum_{j=1}^{k}\sum_{i=1}^{n_{j}}\frac{2i-1}{n_{j}}\left[\frac{\Delta_{3ij}}{F\left(x_{ij};a,b,\beta \right)}-\frac{\Delta_{{3n}_{j}+1-ij}}{1-F\left(x_{n_{j}+1-ij};a,b,\beta \right)}\right]=0,$$

where $\Delta_{1ij},\Delta_{2ij}$ and $\Delta_{3ij}$ are specified in Appendix B.

## Appendix F. The RTAD Equations

$$\frac{\partial R\left(a,b,\beta \right)}{\partial a}=-2\sum_{j=1}^{k}\sum_{i=1}^{n_{j}}\Delta_{1ij}+\sum_{j=1}^{k}\sum_{i=1}^{n_{j}}\frac{2i-1}{n_{j}}\frac{\Delta_{{1n}_{j}+1-ij}}{1-F\left(x_{n_{j}+1-ij};a,b,\beta \right)}=0,$$

$$\frac{\partial R\left(a,b,\beta \right)}{\partial b}=-2\sum_{j=1}^{k}\sum_{i=1}^{n_{j}}\Delta_{2ij}+\sum_{j=1}^{k}\sum_{i=1}^{n_{j}}\frac{2i-1}{n_{j}}\frac{\Delta_{{2n}_{j}+1-ij}}{1-F\left(x_{n_{j}+1-ij};a,b,\beta \right)}=0,$$

$$\frac{\partial R\left(a,b,\beta \right)}{\partial \beta }=-2\sum_{j=1}^{k}\sum_{i=1}^{n_{j}}\Delta_{3ij}+\sum_{j=1}^{k}\sum_{i=1}^{n_{j}}\frac{2i-1}{n_{j}}\frac{\Delta_{{3n}_{j}+1-ij}}{1-F\left(x_{n_{j}+1-ij};a,b,\beta \right)}=0,$$

where $\Delta_{1ij},\Delta_{2ij}$ and $\Delta_{3ij}$ are specified in Appendix B.

## Appendix G. The LTAD Equations

$$\frac{\partial LT\left(a,b,\beta \right)}{\partial a}=2\sum_{j=1}^{k}\sum_{i=1}^{n_{j}}\Delta_{1ij}-\sum_{j=1}^{k}\sum_{i=1}^{n_{j}}\frac{2i-1}{n_{j}}\frac{\Delta_{1ij}}{F\left(x_{ij};a,b,\beta \right)}=0,$$

$$\frac{\partial LT\left(a,b,\beta \right)}{\partial b}=2\sum_{j=1}^{k}\sum_{i=1}^{n_{j}}\Delta_{2ij}-\sum_{j=1}^{k}\sum_{i=1}^{n_{j}}\frac{2i-1}{n_{j}}\frac{\Delta_{2ij}}{F\left(x_{ij};a,b,\beta \right)}=0,$$

$$\frac{\partial LT\left(a,b,\beta \right)}{\partial \beta }=2\sum_{j=1}^{k}\sum_{i=1}^{n_{j}}\Delta_{3ij}-\sum_{j=1}^{k}\sum_{i=1}^{n_{j}}\frac{2i-1}{n_{j}}\frac{\Delta_{3ij}}{F\left(x_{ij};a,b,\beta \right)}=0,$$

where $\Delta_{1ij},\Delta_{2ij}$ and $\Delta_{3ij}$ are specified in Appendix B.

## Appendix H. The ADLTS Equations

$$\frac{\partial LTS\left(a,b,\beta \right)}{\partial a}=2\sum_{j=1}^{k}\sum_{i=1}^{n_{j}}\frac{\Delta_{1ij}}{F\left(x_{ij};a,b,\beta \right)}-\sum_{j=1}^{k}\sum_{i=1}^{n_{j}}\frac{2i-1}{n_{j}}\frac{\Delta_{1ij}}{F{\left(x_{ij};a,b,\beta \right)}^{2}}=0,$$

$$\frac{\partial LTS\left(a,b,\beta \right)}{\partial b}=2\sum_{j=1}^{k}\sum_{i=1}^{n_{j}}\frac{\Delta_{2ij}}{F\left(x_{ij};a,b,\beta \right)}-\sum_{j=1}^{k}\sum_{i=1}^{n_{j}}\frac{2i-1}{n_{j}}\frac{\Delta_{2ij}}{F{\left(x_{ij};a,b,\beta \right)}^{2}}=0,$$

$$\frac{\partial LTS\left(a,b,\beta \right)}{\partial \beta }=2\sum_{j=1}^{k}\sum_{i=1}^{n_{j}}\frac{\Delta_{3ij}}{F\left(x_{ij};a,b,\beta \right)}-\sum_{j=1}^{k}\sum_{i=1}^{n_{j}}\frac{2i-1}{n_{j}}\frac{\Delta_{3ij}}{F{\left(x_{ij};a,b,\beta \right)}^{2}}=0,$$

where $\Delta_{1ij},\Delta_{2ij}$ and $\Delta_{3ij}$ are specified in Appendix B.

## Appendix I. The MPS Equations

$$\frac{\partial M\left(a,b,\beta \right)}{\partial a}=\sum_{j=1}^{k}\sum_{i=1}^{n_{j}+1}\frac{\Delta_{1ij}-\Delta_{1i-1j}}{\left(n_{j}+1\right)D_{ij}\left(a,b,\beta \right)}=0,$$

$$\frac{\partial M\left(a,b,\beta \right)}{\partial b}=\sum_{j=1}^{k}\sum_{i=1}^{n_{j}+1}\frac{\Delta_{2ij}-\Delta_{2i-1j}}{\left(n_{j}+1\right)D_{ij}\left(a,b,\beta \right)}=0,$$

$$\frac{\partial M\left(a,b,\beta \right)}{\partial \beta }=\sum_{j=1}^{k}\sum_{i=1}^{n_{j}+1}\frac{\Delta_{3ij}-\Delta_{3i-1j}}{\left(n_{j}+1\right)D_{ij}\left(a,b,\beta \right)}=0,$$

where $\Delta_{1ij},\Delta_{2ij}$ and $\Delta_{3ij}$ are specified in Appendix B.

## Appendix J. The MSAD Equations

$$\frac{\partial T\left(a,b,\beta \right)}{\partial a}=\sum_{j=1}^{k}\sum_{i=1}^{n_{j}+1}\frac{D_{ij}\left(a,b,\beta \right)-\frac{1}{n_{j}+1}}{\left|D_{ij}\left(a,b,\beta \right)-\frac{1}{n_{j}+1}\right|}\left\{\Delta_{1ij}-\Delta_{1i-1j}\right\}=0,$$

$$\frac{\partial T\left(a,b,\beta \right)}{\partial b}=\sum_{j=1}^{k}\sum_{i=1}^{n_{j}+1}\frac{D_{ij}\left(a,b,\beta \right)-\frac{1}{n_{j}+1}}{\left|D_{ij}\left(a,b,\beta \right)-\frac{1}{n_{j}+1}\right|}\left\{\Delta_{2ij}-\Delta_{2i-1j}\right\}=0,$$

$$\frac{\partial T\left(a,b,\beta \right)}{\partial \beta }=\sum_{j=1}^{k}\sum_{i=1}^{n_{j}+1}\frac{D_{ij}\left(a,b,\beta \right)-\frac{1}{n_{j}+1}}{\left|D_{ij}\left(a,b,\beta \right)-\frac{1}{n_{j}+1}\right|}\left\{\Delta_{3ij}-\Delta_{3i-1j}\right\}=0,$$

where $D_{ij}\left(a,b,\beta \right)\neq \frac{1}{n_{j}+1}.$ $\Delta_{1ij},\Delta_{2ij}$ and $\Delta_{3ij}$ are specified in Appendix B.

## Appendix K. The MSALD Equations

$$\frac{\partial MS\left(a,b,\beta \right)}{\partial a}=\sum_{j=1}^{k}\sum_{i=1}^{n_{j}+1}\frac{\ln D_{ij}\left(a,b,\beta \right)-\ln\frac{1}{n_{j}+1}}{\left|\ln D_{ij}\left(a,b,\beta \right)-\ln\frac{1}{n_{j}+1}\right|}\frac{1}{D_{ij}\left(a,b,\beta \right)}\left\{\Delta_{1ij}-\Delta_{1i-1j}\right\}=0,$$

$$\frac{\partial MS\left(a,b,\beta \right)}{\partial b}=\sum_{j=1}^{k}\sum_{i=1}^{n_{j}+1}\frac{\ln D_{ij}\left(a,b,\beta \right)-\ln\frac{1}{n_{j}+1}}{\left|\ln D_{ij}\left(a,b,\beta \right)-\ln\frac{1}{n_{j}+1}\right|}\frac{1}{D_{ij}\left(a,b,\beta \right)}\left\{\Delta_{2ij}-\Delta_{2i-1j}\right\}=0,$$

$$\frac{\partial MS\left(a,b,\beta \right)}{\partial \beta }=\sum_{j=1}^{k}\sum_{i=1}^{n_{j}+1}\frac{\ln D_{ij}\left(a,b,\beta \right)-\ln\frac{1}{n_{j}+1}}{\left|\ln D_{ij}\left(a,b,\beta \right)-\ln\frac{1}{n_{j}+1}\right|}\frac{1}{D_{ij}\left(a,b,\beta \right)}\left\{\Delta_{3ij}-\Delta_{3i-1j}\right\}=0,$$

where $D_{ij}\left(a,b,\beta \right)\neq \ln\frac{1}{n_{j}+1}.$ $\Delta_{1ij},\Delta_{2ij}$ and $\Delta_{3ij}$ are specified in Appendix B.
