# Supplementary material for: Reliability Inference in Constant Stress Accelerated Life Tests Based on the Exponentiated Half-Logistic Model
Source: Entropy (Basel). 2026 Oct 16;28(10):1140. doi: 10.3390/e28101140 (PMC13703015; doi:10.3390/e28101140)
Supplement: Supplementary file 1 [file entropy-28-01140-s001.zip › entropy-4559101-supplementary.pdf]

Supplementary File:

Table S1. The biases (first row) and the MSEs (second row) of the parameter estimates under stress-dependent scale parameters for $N = 30$.

a	b	Par	ML	LS	WLS	CM	AD	RTAD	LTAD	ADLTS	MPS	MSAD	MSALD
0.2	0.2	a	0.2532	0.0986	0.1419	0.2882	0.1551	0.2434	0.1821	0.1468	-0.0376	-0.0269	-0.0219
			0.4913	0.4975	0.519	0.6897	0.4506	0.6985	0.4834	0.5653	0.3241	0.3821	0.3936
		b	-0.4617	-0.5312	-0.5237	-0.5676	-0.5414	-0.666	-0.4693	-0.3988	-0.4102	-0.3146	-0.3963
			3.8347	4.2510	4.3224	5.2816	3.9722	5.7996	3.9581	4.4297	2.9336	2.6791	3.4600
		β	0.4053	0.1319	0.211	0.4171	0.2136	0.2726	0.3229	0.3717	-0.0349	0.0433	-0.0083
			0.389	0.2851	0.3194	0.5188	0.252	0.3552	0.3989	0.7115	0.1659	0.2924	0.2056
		α_1	0.3233	0.1127	0.1834	0.4304	0.1471	0.314	0.2242	0.2386	-0.0855	-0.0067	-0.0491
			0.5208	0.8868	1.2347	2.0650	0.3201	1.1112	0.5097	1.0196	0.1705	0.4045	0.2176
		α_2	0.1774	-0.0505	0.0115	0.2222	-0.0084	0.0975	0.079	0.1019	-0.1895	-0.1086	-0.1543
			0.3378	0.3436	0.3926	0.7403	0.2169	0.5632	0.3097	0.5045	0.1758	0.2743	0.2007
0.2	0.5	a	0.2574	0.139	0.1764	0.3296	0.1848	0.314	0.1829	0.1191	-0.0181	-0.0013	-0.0241
			0.5251	0.56	0.5755	0.7775	0.5062	0.8196	0.5036	0.5442	0.3545	0.4168	0.438
		b	-0.508	-0.7061	-0.6856	-0.726	-0.6769	-0.8759	-0.533	-0.3645	-0.5413	-0.4439	-0.4678
			4.1686	4.8908	4.9012	6.0972	4.5028	6.7440	4.2568	4.5161	3.3354	3.4343	3.9185
		β	0.3905	0.1199	0.1932	0.4008	0.2021	0.2802	0.2929	0.341	-0.0348	0.0221	-0.0209
			0.3632	0.2595	0.3	0.4833	0.2391	0.3516	0.3611	0.6403	0.1532	0.2868	0.1936
		α_1	0.3408	0.1297	0.1964	0.4745	0.1712	0.4098	0.2216	0.2087	-0.0952	-0.0112	-0.0679
			0.6317	0.7081	0.8346	1.6640	0.4454	1.6391	0.568	0.8067	0.2072	0.4174	0.2695
		α_2	0.1745	-0.1005	-0.0162	0.223	-0.0425	0.1069	0.0511	0.0982	-0.2552	-0.1523	-0.2164
			0.3874	0.3989	1.1429	1.1560	0.2739	0.943	0.3677	0.6415	0.2251	0.3418	0.239
0.2	0.8	a	0.2225	0.0911	0.1354	0.2746	0.1336	0.25	0.1449	0.0974	-0.0591	0.0133	-0.0461
			0.4968	0.4812	0.493	0.6723	0.4388	0.7394	0.4638	0.5714	0.353	0.4165	0.4494
		b	-0.4031	-0.5905	-0.5872	-0.5616	-0.5446	-0.7044	-0.4419	-0.2803	-0.4462	-0.5037	-0.4185
			4.2287	4.5586	4.5749	5.6542	4.2367	6.4614	4.1332	4.8065	3.3706	3.8958	4.1787
		β	0.3735	0.1043	0.1744	0.3794	0.1839	0.2518	0.2742	0.3493	-0.0433	0.0167	-0.0218
			0.3402	0.2501	0.2565	0.4672	0.2215	0.2981	0.3562	0.6529	0.1435	0.2382	0.1747
		α_1	0.3317	0.0719	0.1382	0.4289	0.1256	0.3518	0.1932	0.2217	-0.1335	-0.0199	-0.0898
			0.6327	0.4176	0.4585	1.0833	0.3399	1.2533	0.4879	0.8224	0.2115	0.3904	0.2946
		α_2	0.2337	-0.0951	-0.0284	0.3153	-0.0295	0.1372	0.074	0.1645	-0.2839	-0.1742	-0.2347
			0.701	0.6965	0.7136	2.2652	0.4464	1.1324	0.6326	1.1922	0.3321	0.5176	0.3815

Table S1. (Continued)

a	b	Par	ML	LS	WLS	CM	AD	RTAD	LTAD	ADLTS	MPS	MSAD	MSALD
0.5	0.2	a	0.3035	0.1654	0.224	0.3905	0.216	0.3587	0.2214	0.1664	-0.0041	-0.0681	-0.0255
			0.5461	0.5449	0.5914	0.8122	0.4881	0.8026	0.5164	0.6127	0.3615	0.3685	0.4169
		b	-0.6294	-0.8085	-0.8218	-0.8786	-0.7871	-10.172	-0.6513	-0.4938	-0.6244	-0.2916	-0.5086
			4.4367	4.9283	5.0753	6.1855	4.5614	6.7504	4.5028	5.1215	3.5419	2.4186	3.8212
		β	0.369	0.1074	0.1872	0.3793	0.1842	0.261	0.2727	0.3264	-0.0456	0.0104	-0.0266
			0.3075	0.2041	0.2346	0.4034	0.1893	0.309	0.2877	0.5888	0.1331	0.2422	0.1562
		α_1	0.4752	0.1784	0.3021	0.7082	0.2263	0.5682	0.3091	0.3367	-0.1242	-0.0441	-0.1055
			1.0636	1.4409	2.5191	4.2638	0.6775	3.6086	1.0868	2.1517	0.3556	1.8824	0.4209
		α_2	0.2165	-0.1355	-0.052	0.3106	-0.0644	0.1792	0.0495	0.127	-0.3308	-0.1871	-0.2812
			0.6853	0.6246	0.6326	2.9736	0.4555	4.4162	0.5475	1.0595	0.3646	0.5723	0.3891
0.5	0.8	a	0.3435	0.185	0.2337	0.4011	0.2398	0.3603	0.2549	0.2202	0.018	0.0406	0.022
			0.617	0.5934	0.6092	0.865	0.5437	0.9027	0.5505	0.649	0.3856	0.4722	0.4882
		b	-0.7115	-0.8762	-0.8572	-0.8844	-0.8556	-1.0420	-0.7157	-0.6044	-0.7268	-0.6144	-0.667
			4.8573	5.2360	5.2582	6.4463	4.9657	7.2058	4.7634	5.2087	3.7663	4.1636	4.7323
		β	0.3718	0.1171	0.1884	0.3783	0.1895	0.2479	0.2867	0.3366	-0.0346	0.0292	-0.0182
			0.3153	0.2159	0.2352	0.4029	0.2	0.2985	0.3103	0.5341	0.1343	0.2257	0.162
		α_1	0.6212	0.23	0.3413	0.8269	0.3001	0.7057	0.4086	0.4692	-0.1221	0.0154	-0.0742
			1.6578	1.4904	1.7384	3.9536	1.0442	5.4543	1.2465	2.3625	0.5121	0.992	0.5876
		α_2	0.3268	-0.1715	-0.0447	0.4032	-0.0676	0.1954	0.1259	0.2282	-0.438	-0.2493	-0.3599
			1.4807	1.0033	1.3449	2.8877	0.8913	3.6418	1.3753	2.5653	0.6907	1.1793	0.8335
0.8	0.8	a	0.352	0.1969	0.2548	0.4472	0.2447	0.4213	0.2557	0.2027	-6.90E+00	0.015	-0.0163
			0.605	0.6027	0.6398	0.9379	0.5331	1.0309	0.5361	0.6318	0.3669	0.4809	0.4399
		b	-0.7275	-0.9758	-0.9561	-1.0078	-0.9223	-1.2055	-0.7774	-0.6059	-0.7577	-0.6108	-0.6226
			4.2725	5.1228	5.0905	6.3763	4.6721	7.6068	4.3116	4.3344	3.4089	3.6554	3.8591
		β	0.3744	0.1233	0.1973	0.3793	0.1919	0.2657	0.2687	0.311	-0.0249	0.0428	-9.17E+00
			0.3099	0.2162	0.2399	0.4083	0.1939	0.3008	0.2811	0.4662	0.1277	0.2332	0.1491
		α_1	0.9066	0.3713	0.5718	1.4175	0.4179	1.2296	0.564	0.7023	-0.205	0.0368	-0.1508
			3.8683	5.0051	6.3057	20.9742	2.3728	19.8122	2.8843	14.2079	1.0879	3.2332	1.2620
		α_2	0.4829	-0.2047	-0.0262	0.7133	-0.1228	0.2728	0.1941	0.4232	-0.6478	-0.316	-0.5276
			3.6684	5.4793	5.4417	15.6312	1.9262	5.2979	12.6168	47.4583	1.5212	5.7038	1.5473

Table S2. The biases (first row) and the MSEs (second row) of the parameter estimates under stress-dependent scale parameters for $N = 60$.

a	b	Par	ML	LS	WLS	CM	AD	RTAD	LTAD	ADLTS	MPS	MSAD	MSALD
0.2	0.2	a	0.1799	0.1511	0.1769	0.2428	0.1585	0.226	0.1538	0.0625	0.0075	0.0016	-0.0013
			0.2309	0.2682	0.2681	0.3318	0.2346	0.3499	0.2423	0.2502	0.1769	0.2133	0.2237
		b	-0.442	-0.6068	-0.5818	-0.6288	-0.5659	-0.7227	-0.467	-0.3238	-0.4372	-0.3416	-0.3783
			1.9362	2.4120	2.3128	2.6723	2.1598	3.0942	2.0585	2.0853	1.7094	1.8106	2.0960
		β	0.2977	0.1794	0.2357	0.3156	0.2129	0.2354	0.2718	0.2107	0.0292	0.0709	0.0379
			0.1902	0.1638	0.1809	0.2475	0.1523	0.1795	0.2203	0.294	0.082	0.1611	0.1054
		α_1	0.1625	0.0898	0.1295	0.22	0.1008	0.171	0.1256	0.0512	-0.0669	-0.0342	-0.0552
			0.1539	0.1673	0.1753	0.2631	0.1358	0.2369	0.1603	0.1687	0.0852	0.1415	0.1055
		α_2	0.0416	-0.0726	-0.0302	0.0401	-0.0483	-0.0289	-0.0022	-0.0372	-0.177	-0.1223	-0.1512
			0.1193	0.1313	0.1262	0.1679	0.1143	0.152	0.1248	0.1475	0.102	0.145	0.1172
0.2	0.5	a	0.143	0.1222	0.1484	0.2116	0.1392	0.2201	0.1247	0.0217	-0.0241	0.0071	-0.0115
			0.2115	0.2586	0.2594	0.3159	0.2346	0.364	0.2299	0.2361	0.172	0.2334	0.2231
		b	-0.3628	-0.59	-0.5545	-0.5952	-0.5534	-0.755	-0.4238	-0.2256	-0.3779	-0.3762	-0.3749
			1.8704	2.4406	2.3539	2.6838	2.2410	3.3326	2.0595	2.0730	1.7093	2.1379	2.1208
		β	0.282	0.1514	0.2135	0.2847	0.1944	0.22	0.2524	0.2097	0.0242	0.0672	0.0341
			0.1672	0.1337	0.1514	0.2058	0.1274	0.16	0.1886	0.2869	0.0705	0.1362	0.0913
		α_1	0.1369	0.0547	0.0994	0.1903	0.0802	0.1629	0.0989	0.0229	-0.0994	-0.042	-0.0732
			0.1438	0.155	0.1639	0.241	0.1349	0.259	0.1498	0.1734	0.0882	0.1415	0.1137
		α_2	0.0352	-0.1173	-0.0647	0.0129	-0.0797	-0.061	-0.0239	-0.0378	-0.2111	-0.153	-0.1862
			0.1284	0.1449	0.133	0.175	0.1215	0.1861	0.1355	0.2305	0.1242	0.1688	0.1355
0.2	0.8	a	0.1903	0.1621	0.1885	0.2522	0.171	0.2505	0.1667	0.0696	0.0122	0.048	0.0218
			0.2287	0.2562	0.255	0.3203	0.2255	0.3512	0.2372	0.2564	0.1644	0.2375	0.2185
		b	-0.465	-0.6708	-0.6429	-0.6646	-0.6209	-0.8084	-0.504	-0.3096	-0.4609	-0.4301	-0.4308
			1.9320	2.4430	2.3215	2.6652	2.1839	3.2292	2.0554	2.1790	1.6459	2.2612	2.1630
		β	0.2907	0.1658	0.2218	0.2983	0.2011	0.2269	0.2692	0.2375	0.0338	0.0987	0.0548
			0.1699	0.1399	0.1533	0.2155	0.1315	0.1657	0.1925	0.2907	0.0733	0.1321	0.0908
		α_1	0.1899	0.096	0.1409	0.2495	0.1121	0.2063	0.1481	0.075	-0.0776	-0.0017	-0.0485
			0.1916	0.2023	0.2068	0.327	0.1588	0.3323	0.1989	0.2276	0.105	0.1695	0.1261
		α_2	0.0518	-0.1119	-0.058	0.0474	-0.0777	-0.0415	-0.0058	-0.018	-0.2333	-0.1385	-0.1916
			0.171	0.2189	0.2038	0.2836	0.1705	0.312	0.1936	0.284	0.1576	0.2249	0.18

Table S2. (Continued)

a	b	Par	ML	LS	WLS	CM	AD	RTAD	LTAD	ADLTS	MPS	MSAD	MSALD
0.5	0.2	a	0.2442	0.216	0.2489	0.3221	0.2287	0.3327	0.2171	0.1159	0.0597	0.0212	0.0222
			0.2829	0.3166	0.3251	0.4056	0.284	0.466	0.2806	0.3037	0.1992	0.2409	0.2515
		b	-0.6059	-0.82	-0.8009	-0.8525	-0.7826	-10.225	-0.6661	-0.4473	-0.5991	-0.4028	-0.4705
			2.2080	2.9137	2.8276	3.2316	2.6336	3.9785	2.3615	2.2894	1.9314	1.8680	2.2271
		β	0.2973	0.1731	0.2275	0.3026	0.2089	0.2402	0.2575	0.2299	0.0514	0.0857	0.0561
			0.1721	0.1324	0.1467	0.2072	0.1273	0.1649	0.1797	0.2869	0.0728	0.1328	0.0935
		α_1	0.2905	0.1716	0.2369	0.3804	0.1912	0.3488	0.2195	0.146	-0.0504	-0.0222	-0.0562
			0.387	0.4031	0.4372	0.6752	0.3122	0.811	0.3566	0.4984	0.192	0.324	0.2405
		α_2	0.0516	-0.1243	-0.0639	0.0467	-0.0938	-0.0545	-0.033	-0.0338	-0.2582	-0.1758	-0.2273
			0.1943	0.3558	0.3133	0.4956	0.202	0.3258	0.2014	0.306	0.1849	0.2407	0.2075
0.5	0.8	a	0.2417	0.2164	0.2462	0.3214	0.2244	0.3334	0.2117	0.1097	0.0592	0.0393	0.0458
			0.2716	0.3144	0.316	0.4038	0.2794	0.4561	0.2677	0.2663	0.194	0.2481	0.2494
		b	-0.6158	-0.8603	-0.8269	-0.8656	-0.8048	-1.0356	-0.677	-0.4633	-0.6489	-0.5099	-0.5778
			2.2235	3.0092	2.8598	3.2875	2.7073	3.9531	2.4023	2.1963	2.0866	2.3624	2.4266
		β	0.2761	0.1546	0.2083	0.2809	0.1875	0.2268	0.2305	0.2019	0.0398	0.0681	0.0416
			0.1518	0.1171	0.1315	0.1843	0.1123	0.1474	0.1565	0.2313	0.0657	0.1206	0.0831
		α_1	0.3163	0.1758	0.2475	0.4199	0.1982	0.3889	0.2287	0.1305	-0.0796	-0.0373	-0.0612
			0.4776	0.4664	0.4955	0.7981	0.388	0.9329	0.4346	0.4957	0.2251	0.3599	0.2942
		α_2	0.0612	-0.19	-0.107	0.0464	-0.1384	-0.0652	-0.0536	-0.0639	-0.361	-0.2532	-0.319
			0.3757	0.4387	0.3979	0.5677	0.3693	0.6444	0.3853	0.527	0.3444	0.5509	0.3781
0.8	0.8	a	0.2741	0.2436	0.2763	0.3608	0.2566	0.366	0.2441	0.1284	0.0586	0.0784	0.0636
			0.2934	0.3186	0.3262	0.4198	0.2885	0.4702	0.2878	0.3076	0.187	0.3059	0.2632
		b	-0.7586	-1.0276	-0.9987	-1.0384	-0.9721	-1.1906	-0.8418	-0.5901	-0.7776	-0.7099	-0.7126
			2.4548	3.2043	3.0808	3.4718	2.9206	4.2421	2.5991	2.4146	2.1628	2.8890	2.7428
		β	0.2761	0.1527	0.2	0.2741	0.1865	0.2253	0.2255	0.1956	0.0385	0.0766	0.0582
			0.1496	0.1101	0.1196	0.1725	0.108	0.1489	0.1411	0.2091	0.0648	0.114	0.0827
		α_1	0.4473	0.2315	0.3316	0.5964	0.2709	0.5416	0.3194	0.1918	-0.1673	-0.0224	-0.0986
			0.9461	0.974	1.0013	1.6993	0.756	1.7971	0.8637	1.1417	0.4348	0.8976	0.5659
		α_2	0.026	-0.3443	-0.236	-0.0019	-0.2679	-0.1414	-0.1546	-0.151	-0.6069	-0.434	-0.5058
			0.8882	1.0246	0.932	1.3092	0.8586	1.4076	0.8798	1.2378	0.8221	1.0962	0.8829

Table S3. The biases (first row) and the MSEs (second row) of the parameter estimates under stress-dependent scale parameters for $N = 120$.

a	b	Par	ML	LS	WLS	CM	AD	RTAD	LTAD	ADLTS	MPS	MSAD	MSALD
0.2	0.2	a	0.156	0.1714	0.1863	0.2181	0.1732	0.2352	0.151	0.0499	0.0527	0.0612	0.0505
			0.1198	0.1556	0.1568	0.1809	0.1395	0.2123	0.1291	0.1319	0.0921	0.1359	0.1237
		b	-0.4649	-0.6538	-0.6237	-0.6711	-0.6206	-0.7903	-0.511	-0.3271	-0.455	-0.4091	-0.4312
			1.0683	1.5458	1.4585	1.6327	1.3928	2.0464	1.2059	1.1215	1.0063	1.3109	1.2894
		β	0.2456	0.1817	0.2233	0.2481	0.2025	0.2176	0.232	0.1586	0.0961	0.1195	0.0897
			0.1017	0.085	0.0967	0.1167	0.085	0.0994	0.113	0.1693	0.0477	0.0791	0.0562
		α_1	0.0997	0.0755	0.1025	0.1362	0.0825	0.131	0.0828	0.0082	-0.0334	-0.0019	-0.0256
			0.0594	0.0731	0.0767	0.0964	0.0604	0.1101	0.063	0.0773	0.0381	0.0657	0.0505
		α_2	-0.0249	-0.096	-0.0649	-0.0452	-0.08	-0.0796	-0.0527	-0.0794	-0.1483	-0.1079	-0.1354
			0.0382	0.0558	0.0464	0.0551	0.0465	0.0682	0.0451	0.0674	0.0513	0.0657	0.0589
0.2	0.5	a	0.1532	0.1702	0.1847	0.2142	0.1752	0.2427	0.1486	0.0436	0.0533	0.0819	0.0533
			0.1285	0.1506	0.1517	0.1741	0.1413	0.2071	0.1306	0.1436	0.1025	0.153	0.1286
		b	-0.4493	-0.6512	-0.6243	-0.6535	-0.6236	-0.8125	-0.4948	-0.2868	-0.4659	-0.4755	-0.4206
			1.1326	1.5354	1.4499	1.5988	1.4195	2.0159	1.2419	1.1903	1.1002	1.4737	1.3228
		β	0.243	0.1785	0.2163	0.2445	0.2004	0.2137	0.2333	0.1663	0.0985	0.1222	0.0975
			0.1029	0.0869	0.097	0.1184	0.0852	0.0974	0.1181	0.1633	0.0498	0.0826	0.0585
		α_1	0.1086	0.0787	0.1059	0.1435	0.0901	0.1418	0.0903	0.0142	-0.0355	0.0107	-0.0192
			0.0746	0.0783	0.0809	0.1037	0.0692	0.1122	0.0743	0.0985	0.0494	0.0849	0.0617
		α_2	-0.0209	-0.1056	-0.0731	-0.0434	-0.0871	-0.0912	-0.0493	-0.0726	-0.1706	-0.1284	-0.1409
			0.057	0.08	0.0685	0.0809	0.0655	0.0885	0.0732	0.0985	0.0713	0.0883	0.0802
0.2	0.8	a	0.1739	0.1939	0.2106	0.2389	0.1924	0.2727	0.1651	0.0611	0.0653	0.0854	0.0852
			0.126	0.1617	0.1624	0.1875	0.1444	0.2312	0.1286	0.124	0.0933	0.135	0.1234
		b	-0.5147	-0.7357	-0.7012	-0.7379	-0.69	-0.8945	-0.5606	-0.3654	-0.5046	-0.5055	-0.5377
			1.1571	1.6691	1.5517	1.7263	1.4954	2.2351	1.2610	1.1418	1.1043	1.4198	1.3734
		β	0.2511	0.1816	0.2247	0.2458	0.2022	0.2249	0.2289	0.1625	0.1074	0.1302	0.1096
			0.1057	0.0882	0.1005	0.1188	0.0865	0.1064	0.1112	0.1428	0.0515	0.0874	0.0608
		α_1	0.1251	0.0953	0.1298	0.1663	0.1016	0.1757	0.0998	0.0122	-0.0335	0.0056	-0.0102
			0.0791	0.0951	0.1005	0.1264	0.0783	0.1504	0.0795	0.0877	0.0489	0.089	0.0625
		α_2	-0.0308	-0.1325	-0.0882	-0.0616	-0.111	-0.1021	-0.071	-0.1035	-0.1947	-0.1527	-0.1812
			0.08	0.1127	0.0955	0.111	0.0927	0.1314	0.0943	0.1258	0.1023	0.1365	0.1076

Table S3. (Continued)

a	*b*	Par	ML	LS	WLS	CM	AD	RTAD	LTAD	ADLTS	MPS	MSAD	MSALD
0.5	0.2	a	0.1839	0.2008	0.2166	0.2519	0.2006	0.2811	0.1736	0.0628	0.0887	0.0677	0.0567
			0.1279	0.1648	0.1598	0.1947	0.146	0.2357	0.1294	0.1314	0.0945	0.13	0.1263
		b	-0.5286	-0.7279	-0.701	-0.7416	-0.6923	-0.8881	-0.5801	-0.3816	-0.5738	-0.4473	-0.4517
			1.0969	1.5452	1.4366	1.6211	1.3938	2.0768	1.1757	1.1350	1.0819	1.2144	1.2830
		β	0.2508	0.1887	0.2265	0.2518	0.2053	0.2291	0.2213	0.147	0.1086	0.1216	0.1021
			0.0997	0.0838	0.0937	0.1144	0.0816	0.1028	0.0989	0.1258	0.0489	0.0721	0.0556
		α_1	0.1651	0.1328	0.1673	0.2265	0.1381	0.2335	0.1299	0.016	-0.0215	-0.0016	-0.0278
			0.1278	0.158	0.1523	0.2149	0.1285	0.2508	0.1273	0.1406	0.0819	0.1308	0.1022
		α_2	-0.0262	-0.127	-0.0864	-0.048	-0.108	-0.0931	-0.0769	-0.1176	-0.2137	-0.1573	-0.1825
			0.0822	0.1117	0.0925	0.1127	0.0918	0.1313	0.0935	0.1418	0.115	0.1313	0.1201
0.5	0.8	a	0.2253	0.2403	0.2568	0.29	0.2433	0.3214	0.22	0.1071	0.1049	0.1239	0.1078
			0.1544	0.1809	0.1845	0.2156	0.1683	0.2635	0.1539	0.1489	0.1034	0.1595	0.1385
		b	-0.6563	-0.8692	-0.8366	-0.8705	-0.8336	-1.0336	-0.7167	-0.5057	-0.6585	-0.6197	-0.6183
			1.3191	1.7964	1.7032	1.8673	1.6576	2.3952	1.4177	1.2951	1.2155	1.6252	1.5169
		β	0.2545	0.1921	0.2266	0.2522	0.2099	0.2259	0.231	0.1646	0.1019	0.1388	0.1186
			0.1004	0.0813	0.0908	0.1106	0.0809	0.0983	0.0983	0.1187	0.0404	0.077	0.0567
		α_1	0.2231	0.1732	0.2184	0.2825	0.1871	0.2905	0.188	0.0604	-0.0245	0.0456	0.0037
			0.1965	0.2111	0.2191	0.2925	0.1823	0.3551	0.1862	0.2142	0.1063	0.2018	0.1391
		α_2	-0.0525	-0.1936	-0.1372	-0.0873	-0.1637	-0.1527	-0.1134	-0.1597	-0.3072	-0.2166	-0.261
			0.1468	0.2036	0.167	0.1997	0.168	0.2353	0.1638	0.2415	0.201	0.2684	0.2161
0.8	0.8	a	0.2378	0.2696	0.2878	0.3272	0.2642	0.3605	0.2332	0.1032	0.1127	0.1254	0.117
			0.1636	0.2183	0.2192	0.261	0.1894	0.3112	0.1695	0.1481	0.1174	0.1648	0.1408
		b	-0.6927	-0.9783	-0.9302	-0.9831	-0.9221	-1.1560	-0.775	-0.4952	-0.7201	-0.6844	-0.6804
			1.3914	2.1175	1.9553	2.1915	1.8853	2.7619	1.5740	1.2475	1.4172	1.7270	1.5881
		β	0.2493	0.1889	0.226	0.2489	0.2024	0.225	0.2228	0.1718	0.1116	0.1293	0.1176
			0.094	0.0797	0.0897	0.1087	0.0752	0.096	0.0915	0.1156	0.0412	0.0666	0.051
		α_1	0.3184	0.2699	0.3428	0.4424	0.2672	0.453	0.2643	0.0839	-0.0423	0.0322	-0.002
			0.3794	0.5096	0.5234	0.7185	0.3729	0.8589	0.3782	0.4286	0.2052	0.3505	0.2542
		α_2	-0.0748	-0.291	-0.1968	-0.1283	-0.2575	-0.2235	-0.1757	-0.2039	-0.4576	-0.3608	-0.3926
			0.2768	0.4471	0.3491	0.4485	0.3428	0.5076	0.3406	0.4969	0.4028	0.4914	0.4186

Additional simulation experiments were conducted to assess the robustness of the estimation procedures under unequal group allocations. The corresponding results for the considered sample sizes and allocation schemes are reported in the tables below.

Table S4. The biases (first row) and the MSEs (second row) of the estimators for $n_1 = 20, n_2 = 40$ under two accelerated stress levels.

a	b	Par	ML	LS	WLS	CM	AD	RTAD	LTAD	ADLTS	MPS	MSAD	MSALD
0.2	0.5	a	0.0716	-0.0187	0.0139	0.0701	0.0069	0.0555	0.0126	-0.0568	-0.1345	-0.0884	-0.1483
			0.2732	0.3323	0.3388	0.3731	0.2915	0.4105	0.2846	0.3211	0.2484	0.1578	0.2073
		b	-0.0594	-0.0409	-0.0327	-0.0428	-0.0336	-0.0992	0.0054	0.0836	0.0588	-0.0524	0.1039
			2.1792	2.6445	2.5837	2.9357	2.3842	3.2495	2.3019	2.4582	1.8722	0.8334	1.4193
		β	0.0662	-0.0483	0.0019	0.0703	-0.0116	0.0151	0.0250	-0.0107	-0.1588	-0.1507	-0.1589
			0.0766	0.0895	0.0882	0.1026	0.0722	0.0945	0.0921	0.2066	0.0855	0.1265	0.1057
		α_1	0.1348	0.0276	0.0781	0.1628	0.0529	0.1341	0.0729	0.0155	-0.1187	-0.0818	-0.1237
			0.1901	0.2078	0.2395	0.3077	0.1676	0.3395	0.1814	0.2230	0.1152	0.1357	0.1185
		α_2	0.1084	-0.0065	0.0434	0.1353	0.0273	0.0827	0.0617	0.0233	-0.1299	-0.1146	-0.1168
			0.1346	0.1525	0.1491	0.2221	0.1237	0.2238	0.1386	0.2000	0.0953	0.1329	0.1143
0.2	0.8	a	0.0777	-0.0234	0.0088	0.0643	0.0027	0.0449	0.0146	-0.0403	-0.1250	-0.0717	-0.1284
			0.2617	0.3151	0.3212	0.3532	0.2813	0.4062	0.2679	0.2936	0.2358	0.1472	0.2106
		b	-0.0723	-0.0393	-0.0222	-0.0272	-0.0338	-0.0915	0.0115	0.0580	0.0186	-0.0760	0.0307
			2.0669	2.4026	2.3360	2.6656	2.2009	3.1541	2.0980	2.4016	1.7741	0.8011	1.4952
		β	0.0714	-0.0406	0.0100	0.0768	-0.0070	0.0127	0.0417	0.0082	-0.1514	-0.1255	-0.1527
			0.0695	0.0845	0.0856	0.0994	0.0669	0.0817	0.0932	0.1795	0.0762	0.1224	0.0972
		α_1	0.1496	0.0247	0.0815	0.1709	0.0528	0.1293	0.0844	0.0278	-0.1235	-0.0695	-0.1223
			0.2247	0.2401	0.2842	0.3572	0.2026	0.3602	0.2162	0.2439	0.1336	0.1695	0.1413
		α_2	0.1286	-0.0120	0.0520	0.1564	0.0250	0.0820	0.0824	0.0457	-0.1535	-0.1128	-0.1418
			0.1973	0.1951	0.2069	0.2903	0.1599	0.2863	0.2028	0.3219	0.1355	0.1986	0.1676
0.5	0.8	a	0.0578	-0.0565	-0.0213	0.0401	-0.0286	0.0097	-0.0088	-0.0650	-0.1615	-0.1016	-0.1368
			0.2675	0.3325	0.3428	0.3680	0.2840	0.4234	0.2713	0.3046	0.2520	0.1792	0.2081
		b	-0.0251	0.0331	0.0352	0.0500	0.0398	0.0279	0.0506	0.0911	0.0621	-0.0489	0.0003
			1.9811	2.4168	2.3618	2.6925	2.1520	3.0763	2.0610	2.2268	1.6886	0.8410	1.3745
		β	0.0522	-0.0522	-0.0117	0.0592	-0.0201	0.0084	0.0153	-0.0172	-0.1599	-0.1267	-0.1556
			0.0581	0.0713	0.0680	0.0802	0.0573	0.0833	0.0693	0.1526	0.0725	0.1140	0.0862
		α_1	0.1895	0.0103	0.0899	0.2289	0.0446	0.1874	0.0864	0.0160	-0.2085	-0.1205	-0.1877
			0.4783	0.5420	0.7019	0.8204	0.4065	1.1362	0.4196	0.5011	0.2883	0.4323	0.2887
		α_2	0.1708	-0.0182	0.0560	0.2372	0.0342	0.1711	0.0911	0.0398	-0.2466	-0.1842	-0.2367
			0.3482	0.4250	0.3988	0.6571	0.3235	0.9215	0.3487	0.5979	0.2507	0.3865	0.2883

Table S5. The biases (first row) and the MSEs (second row) of the estimators for $n_1 = 10, n_2 = 20$ under two accelerated stress levels.

a	b	Par	ML	LS	WLS	CM	AD	RTAD	LTAD	ADLTS	MPS	MSAD	MSALD
0.2	0.5	a	0.2020	-0.0164	0.0346	0.1741	0.0373	0.1374	0.0669	0.0496	-0.1438	-0.1053	-0.1524
			0.6353	0.6384	0.6989	0.8327	0.5778	0.9225	0.5887	0.6763	0.4811	0.2716	0.3235
		b	-0.2245	-0.1125	-0.1186	-0.1311	-0.1017	-0.2083	-0.0513	-0.0182	-0.0318	-0.0914	-0.0097
			4.4872	4.6069	4.8227	5.7552	4.3578	6.6359	4.3446	5.0321	3.4233	1.2587	2.0496
		β	0.1901	-0.0534	0.0158	0.1970	0.0210	0.0819	0.1144	0.1639	-0.1950	-0.1591	-0.1990
			0.2185	0.2127	0.2079	0.3085	0.1726	0.2396	0.2587	0.4968	0.1713	0.2505	0.2017
		α_1	0.3809	0.0987	0.1899	0.4522	0.1491	0.3978	0.2200	0.2429	-0.1026	-0.0556	-0.1187
			0.8876	0.6761	0.9082	1.6729	0.5267	1.9935	0.6762	0.9451	0.2910	0.4805	0.2612
		α_2	0.2673	0.0096	0.0760	0.3407	0.0769	0.2511	0.1688	0.2094	-0.1611	-0.1067	-0.1493
			0.4674	0.4136	0.4185	0.9252	0.3200	1.1030	0.4706	0.7863	0.2016	0.4204	0.2358
0.2	0.8	a	0.1423	-0.0658	-0.0157	0.1139	-0.0186	0.0711	0.0093	-0.0064	-0.1924	-0.1441	-0.2001
			0.5725	0.6576	0.7335	0.8303	0.5498	0.9195	0.5444	0.6615	0.4661	0.2251	0.3682
		b	-0.1032	-0.0552	-0.0496	-0.0340	-0.0143	-0.0856	0.0321	0.1109	0.0386	-0.0560	0.0675
			4.3816	4.9596	5.2118	6.1949	4.4404	7.0996	4.3172	4.9454	3.3804	1.0283	2.2572
		β	0.1301	-0.1145	-0.0458	0.1222	-0.0385	0.0194	0.0424	0.1226	-0.2378	-0.2101	-0.2379
			0.1806	0.1902	0.1916	0.2392	0.1525	0.1941	0.2132	0.4531	0.1758	0.2196	0.2109
		α_1	0.3284	0.0523	0.1756	0.4109	0.0882	0.3513	0.1533	0.2248	-0.1604	-0.1248	-0.1559
			0.8260	0.8491	1.8430	2.0950	0.4919	2.4013	0.5945	1.3959	0.3005	0.3195	0.4227
		α_2	0.2879	-0.0322	0.0550	0.3426	0.0545	0.2538	0.1482	0.2441	-0.2070	-0.1669	-0.1898
			0.6368	0.4809	0.5368	1.0786	0.4080	1.3519	0.5283	1.1976	0.2816	0.4233	0.3353
0.5	0.8	a	0.1734	-0.0345	0.0263	0.1730	0.0129	0.1389	0.0403	0.0196	-0.1963	-0.1718	-0.2070
			0.6184	0.6551	0.7512	0.8540	0.5647	1.0312	0.5812	0.7279	0.4877	0.2626	0.3149
		b	-0.1374	-0.1316	-0.1265	-0.1157	-0.0917	-0.2272	-0.0189	0.0864	0.0079	-0.0433	0.0336
			4.5888	4.8774	5.0792	6.1082	4.4838	7.3054	4.4433	5.4431	3.4648	1.1157	1.7340
		β	0.1416	-0.0851	-0.0131	0.1466	-0.0204	0.0441	0.0587	0.1246	-0.2163	-0.1979	-0.2222
			0.1637	0.1789	0.1895	0.2410	0.1366	0.2029	0.1884	0.3844	0.1520	0.2033	0.1787
		α_1	0.5272	0.1195	0.3400	0.6960	0.1717	0.8781	0.2822	0.3997	-0.2225	-0.1810	-0.2469
			1.8941	1.6106	4.6834	4.3011	1.1596	44.6755	1.5257	3.6757	0.5997	1.0266	0.5328
		α_2	0.4593	0.0025	0.1495	0.6196	0.0994	0.4704	0.2513	0.4561	-0.3001	-0.2366	-0.2933
			1.4031	1.3104	1.4941	3.5852	0.9263	4.6745	1.2165	5.7274	0.5573	1.3895	0.5906

Table S6. The biases (first row) and the MSEs (second row) of the estimators under three accelerated stress levels.

							$n_1 = 10, n_2 = 20, n_3 = 30$						
a	b	Par	ML	LS	WLS	CM	AD	RTAD	LTAD	ADLTS	MPS	MSAD	MSALD
0.2	0.5	a	0.0785	-0.1079	-0.0195	0.0191	-0.0654	0.0061	-0.0745	-0.2231	-0.1834	-0.1087	-0.1742
			0.2153	0.2537	0.3305	0.2833	0.2199	0.3174	0.2151	0.2984	0.2240	0.2895	0.2545
		b	-0.0406	-0.0104	-0.0060	-0.0036	0.0031	-0.0924	0.0672	0.2415	0.0094	-0.0800	0.0441
			0.8187	0.9568	1.1688	1.1176	0.8705	1.2461	0.8240	0.9236	0.7399	1.1020	0.8954
		β	0.0824	-0.1408	-0.0213	0.0275	-0.0793	-0.0540	-0.0407	-0.1184	-0.2364	-0.1928	-0.1961
			0.0722	0.1011	0.0870	0.0984	0.0721	0.0848	0.0930	0.1826	0.1175	0.1682	0.1033
		α_1	0.1697	-0.0683	0.0852	0.1225	-0.0213	0.0899	-0.0155	-0.1422	-0.1733	-0.0751	-0.1457
			0.2963	0.2501	0.4925	0.4148	0.2060	0.4502	0.2103	0.2376	0.1737	0.2783	0.2019
		α_2	0.1278	-0.1227	0.0136	0.0737	-0.0634	0.0036	-0.0363	-0.1325	-0.2216	-0.1510	-0.1887
			0.1311	0.1251	0.1645	0.1757	0.0963	0.1736	0.1075	0.1527	0.1148	0.1628	0.1154
		α_3	0.1380	-0.1235	0.0163	0.1006	-0.0558	-0.0084	-0.0051	-0.0623	-0.2338	-0.1711	-0.1848
			0.1980	0.2087	0.2075	0.3157	0.1625	0.2746	0.1831	0.2201	0.1609	0.2697	0.1634
							$n_1 = 20, n_2 = 30, n_3 = 40$						
a	b	Par	ML	LS	WLS	CM	AD	RTAD	LTAD	ADLTS	MPS	MSAD	MSALD
0.2	0.5	a	0.0286	-0.0919	-0.0322	-0.0110	-0.0600	-0.0344	-0.0547	-0.1565	-0.1533	-0.1323	-0.1472
			0.1123	0.1375	0.1467	0.1426	0.1204	0.1600	0.1196	0.1688	0.1207	0.1701	0.1455
		b	0.0092	0.0187	0.0243	0.0324	0.0286	0.0053	0.0456	0.0924	0.0303	0.0147	0.0397
			0.4578	0.5099	0.5518	0.5652	0.4777	0.6491	0.4539	0.5099	0.3995	0.6206	0.5203
		β	0.0451	-0.1071	-0.0248	0.0063	-0.0607	-0.0461	-0.0368	-0.1520	-0.1838	-0.1615	-0.1683
			0.0448	0.0646	0.0548	0.0596	0.0477	0.0560	0.0601	0.1423	0.0706	0.0931	0.0734
		α_1	0.0774	-0.0752	0.0098	0.0405	-0.0367	0.0054	-0.0235	-0.1288	-0.1586	-0.1195	-0.1423
			0.1187	0.1148	0.1428	0.1458	0.1006	0.1514	0.1091	0.1392	0.0936	0.1433	0.1087
		α_2	0.0677	-0.0982	-0.0090	0.0301	-0.0518	-0.0189	-0.0326	-0.1381	-0.1821	-0.1522	-0.1661
			0.0663	0.0767	0.0765	0.0891	0.0593	0.0902	0.0661	0.1055	0.0727	0.0957	0.0767
		α_3	0.0878	-0.0925	0.0061	0.0567	-0.0384	-0.0038	-0.0129	-0.1167	-0.1850	-0.1517	-0.1620
			0.1204	0.1278	0.1252	0.1619	0.1064	0.1720	0.1104	0.1517	0.1069	0.1503	0.1188
							$n_1 = 30, n_2 = 40, n_3 = 50$						
a	b	Par	ML	LS	WLS	CM	AD	RTAD	LTAD	ADLTS	MPS	MSAD	MSALD
0.2	0.5	a	0.0270	-0.0600	-0.0077	0.0002	-0.0359	-0.0168	-0.0316	-0.1272	-0.1139	-0.0724	-0.1102
			0.0759	0.0923	0.0933	0.0956	0.0808	0.1149	0.0777	0.1215	0.0812	0.1107	0.1034
		b	-0.0168	-0.0282	-0.0198	-0.0171	-0.0207	-0.0400	-0.0039	0.0510	-0.0099	-0.0544	-0.0016
			0.3419	0.3874	0.3870	0.4180	0.3591	0.5023	0.3360	0.3979	0.3080	0.4777	0.4325
		β	0.0305	-0.0891	-0.0152	-0.0039	-0.0543	-0.0444	-0.0324	-0.1373	-0.1533	-0.1220	-0.1407
			0.0326	0.0468	0.0375	0.0424	0.0353	0.0412	0.0451	0.1263	0.0520	0.0695	0.0547
		α_1	0.0549	-0.0600	0.0126	0.0252	-0.0305	0.0016	-0.0201	-0.1146	-0.1296	-0.0772	-0.1177
			0.0699	0.0755	0.0828	0.0891	0.0634	0.1034	0.0647	0.1051	0.0629	0.0913	0.0748
		α_2	0.0431	-0.0875	-0.0081	0.0074	-0.0521	-0.0285	-0.0350	-0.1255	-0.1556	-0.1149	-0.1432
			0.0417	0.0533	0.0474	0.0560	0.0398	0.0621	0.0426	0.0888	0.0524	0.0691	0.0574
		α_3	0.0518	-0.0956	-0.0073	0.0139	-0.0547	-0.0311	-0.0307	-0.1127	-0.1672	-0.1295	-0.1469
			0.0869	0.0970	0.0854	0.1091	0.0798	0.1220	0.0833	0.1387	0.0865	0.1301	0.1038

Table S7. Summary of the Best Estimation Methods Based on Bias (first row) and MSE (second row).

N	a, b	a	b	β	α_1	α_2
30	0.2, 0.2	LS	MSALD	LS	MSALD	MSALD
		MPS	MPS	MPS	MPS	MPS
	0.2, 0.5	WLS	LS	MSAD	MSALD	MSALD
		MPS	MPS	MPS	MPS	MPS
	0.2, 0.8	WLS	LS	LS	MSAD	MSALD
		AD	MPS	MPS	MPS	MPS
	0.5, 0.2	WLS	LS	LS	MSAD	MSAD
		MPS	MPS	MPS	MPS	MPS
	0.5, 0.8	WLS	LS	LS	MSALD	MSALD
		AD	MPS	MPS	MPS	MPS
	0.8, 0.8	LS	MSAD	LS	MSALD	MPS
		MPS	MPS	MPS	MPS	MPS
60	0.2, 0.2	AD	WLS	WLS	LS	LS
		ML	MPS	MPS	MPS	MPS
	0.2, 0.5	WLS	LS	LS	MSAD	MSAD
		MPS	MPS	MPS	MPS	MPS
	0.2, 0.8	ADLTS	WLS	AD	MSAD	MSAD
		AD	MPS	MPS	MPS	MPS
	0.5, 0.2	WLS	LS	LS	MSAD	MSAD
		MPS	MPS	MPS	MPS	MPS
	0.5, 0.8	ADLTS	WLS	WLS	MSAD	MSAD
		AD	MPS	MPS	MPS	MPS
	0.8, 0.8	WLS	LS	LS	MSAD	MSAD
		MPS	MPS	MPS	MPS	MPS
120	0.2, 0.2	WLS	MSALD	LS	ADLTS	ADLTS
		ML	MPS	MPS	MPS	MPS
	0.2, 0.5	WLS	LS	LS	LS	LS
		ML	MPS	MPS	MPS	MPS
	0.2, 0.8	WLS	LS	ADLTS	LS	MSAD
		ML	MPS	MPS	MPS	MPS
	0.5, 0.2	WLS	LS	LS	LS	LS
		ML	ML	ML	MPS	MPS
	0.5, 0.8	LS	LS	ADLTS	MSAD	MSAD
		ML	MPS	ML	MPS	MPS
	0.8, 0.8	LS	LS	LS	MSAD	MSAD
		MPS	MPS	MPS	MPS	MPS

Table S8. Summary of the estimation methods considered in the study.

Estimator	Core Concept	Main Merits	Main Limitations	Application Scenarios
ML	Estimates the parameters by maximizing the likelihood function of the observed sample.	Well-established likelihood-based inference; provides a general framework for parameter estimation	Requires numerical optimization for the EHL-CSALT model; may be affected by model misspecification and optimization issues	General-purpose parameter and reliability inference when the assumed EHL-CSALT model is appropriate
LS	Estimates parameters by minimizing the squared differences between empirical and theoretical quantities.	Simple formulation; familiar and computationally convenient.	Squared deviations can give relatively high weight to large discrepancies and may therefore be affected by extreme observations.	General estimation when a simple distance-based criterion is desired.
WLS	Extends LS by assigning different weights to the observations or fitted quantities.	Allows different observations to contribute with unequal weights; retains the intuitive LS framework	Results depend on the weighting structure; observations with extreme weights may have a substantial effect on estimation	Data with unequal variability or observations having different levels of precision.
CM	Minimizes the integrated squared difference between the empirical and theoretical distribution functions.	Simple distributional goodness-of-fit-based estimation; considers discrepancies across the sample distribution	Does not specifically emphasize either tail; performance may depend on the overall distributional fit	General distributional fitting when overall agreement is of interest.
AD	Minimizes a criterion based on the squared difference between empirical and theoretical distributions with greater emphasis on the tails.	Places relatively greater emphasis on discrepancies in the tails than criteria such as CM; useful for detecting distributional misfit in tail regions	Tail sensitivity can make the estimator more affected by unusual/extreme observations; does not specifically distinguish the left and right tails	Reliability analysis and applications where tail behavior is important.
RTAD	Minimization of a right-tail-sensitive Anderson–Darling-type criterion	Places particular emphasis on the upper/right tail of the distribution; useful when right-tail agreement is important	Greater sensitivity to observations affecting the right tail; may be less informative about left-tail behavior	Reliability and lifetime applications where accurate modeling of upper-tail/larger-lifetime behavior is important
LTAD	Minimization of a left-tail-sensitive Anderson–Darling-type criterion	Places particular emphasis on the lower/left tail of the distribution; useful when lower-tail agreement is important	Greater sensitivity to observations affecting the left tail; may provide less information about right-tail behavior	Reliability and lifetime applications where accurate modeling of lower-tail/early-failure behavior is important
ADLTS	Minimization of an Anderson–Darling-based second-order criterion with emphasis on the left tail	Places greater emphasis on discrepancies in the left tail; can be useful for modeling lower-lifetime behavior	May provide less emphasis on the overall distribution or right-tail behavior; may be sensitive to observations influencing the left tail	Reliability applications where early failures or lower-lifetime behavior are of particular interest
MPS	Maximization of the logarithm of the product of spacings between consecutive ordered observations	Provides an alternative to likelihood-based estimation; can be useful when likelihood-based optimization is difficult or less stable	Performance depends on the spacing structure of the ordered observations; may be sensitive to very small spacings	Reliability and lifetime-data applications where an alternative to maximum likelihood estimation is desired
MSAD	Minimization of the sum of absolute deviations between the observed spacings and their expected equal-spacing values	Uses absolute rather than squared deviations, reducing the influence of the magnitude of individual spacing discrepancies	The absolute-value criterion is non-differentiable when a spacing equals its target value; performance depends on the spacing structure	Reliability and lifetime-data applications where an alternative spacing-based estimation criterion is desired and large spacing discrepancies should not be squared
MSALD	Minimization of the sum of absolute deviations between the logarithms of the observed spacings and the logarithm of their expected equal-spacing value	Combines a spacing-based criterion with a logarithmic transformation and absolute deviations; reduces the emphasis on the magnitude of large spacing discrepancies	The log transformation requires positive spacings; the absolute-value criterion is non-differentiable at the target spacing	Reliability and lifetime-data applications where spacing-based estimation with reduced emphasis on large spacing discrepancies is desired
